# Psychometric Analyses of Commonly Used Patient‐Reported Outcome and Experience Measures in Genetic Counseling Research: Findings From the GC‐PRO Study

**DOI:** 10.1002/jgc4.70300

**Published:** 2026-10-23

**Authors:** Robert F. Dedrick, Heather A. Zierhut, Elena R. Fisher, Deborah Cragun

**Affiliations:** ^1^ Department of Educational and Psychological Studies University of South Florida Tampa Florida USA; ^2^ Department of Genetics, Cell Biology, and Development University of Minnesota Minneapolis Minnesota USA; ^3^ College of Public Health University of South Florida Tampa Florida USA

**Keywords:** confirmatory factor analysis, genetic counseling, genetic counseling research, patient‐reported experience measures, patient‐reported outcome measures, psychometric properties

## Abstract

Patient‐reported outcome measures (PROMs) and patient‐reported experience measures (PREMs) are standardized questionnaires that evaluate patients' outcomes and experiences of health services, respectively. Despite their widespread use in genetic counseling (GC) research, many PROMs and PREMs lack comprehensive evidence of measurement quality, particularly regarding validity, fairness, and reliability. This study evaluated 10 commonly used PROMs/PREMs in Stage 1 of the GC‐PRO study by examining content validity, internal structure, relationships to other variables, fairness, and internal consistency reliability. A total of 453 patients receiving GC services across prenatal, cancer, cardiology, and general genetics specialties completed pre‐ and post‐session surveys that included three PROMs: Genetic Counseling Outcomes Scale (GCOS), Patient Activation Measure (PAM), SURE Decisional Conflict, and seven PREMs: Healthcare Climate Questionnaire (HCCQ), Genetic Counseling Satisfaction Scale (GCSS), Patient‐Centered Communication Scale (PCCS), State‐Based Information Overload, Working Alliance Inventory (WAI), CollaboRATE, and CAHPS Health Literacy Supplemental. Content validity was assessed through expert and community partner interviews. Confirmatory factor analysis evaluated the internal structure of each measure using standard model fit indices (χ^2^, root mean square error of approximation, comparative fit index, and the standardized root mean square residual). Cronbach's alpha was calculated for all multi‐item scales. Five measures (e.g., GCSS, PCCS, HCCQ, WAI, and CollaboRATE) demonstrated generally strong psychometric performance across multiple forms of evidence. Psychometric results for the GCOS, PAM, State‐Based Information Overload, and SURE scale were mixed with some results providing evidence of good measurement quality while other sources of evidence suggested that refinement may be needed. Our multi‐faceted approach to psychometric evaluation of 10 commonly used PROMs/PREMs provides a foundation for improving the quality of these instruments in the GC field. Ultimately, our results argue against a “one‐size‐fits‐all” approach to outcome measurement in GC research.

## Abstract (highlights)

**What Is Known About This Topic:**

- Patient‐reported outcome measures (PROMs) and patient‐reported experience measures (PREMs) are widely used tools that help researchers understand patients' experiences and outcomes, but past reviews have shown that many measures used in genetic counseling (GC) research report only limited evidence of their quality.
- Measurement quality can vary across settings, populations, and uses; therefore, researchers must collect and report on their own evidence of validity, fairness, and reliability when using PROMs/PREMs in GC research.

**What This Paper Adds to the Topic:**

- This study provides a comprehensive psychometric analysis of 10 commonly used PROMs and PREMs in GC research, offering new evidence on validity, reliability, and fairness across diverse patient groups and clinical specialties.
- Through our psychometric validation analyses, this paper highlights that outcome measurement in GC research cannot rely on a single standardized tool and provides guidance to GC researchers on choosing and improving measures in future research.

## Plain Language Summary

This study looked at how well 10 surveys used in genetic counseling research measure patients' experiences and outcomes. Some survey measures worked very well, while others may require more testing or improvement. Measuring the impact of genetic counseling through these survey measures can be challenging.

## 1 Introduction

For nearly half a century, clinical research studies have utilized patient‐reported outcome measures (PROMs), which assess patients' attitudes, capacities, or behaviors, and patient‐reported experience measures (PREMs), which are post‐visit assessments of patients' experiences related to the care they received (Churruca et al. 2021). These measures have been used to assess the effectiveness of health‐related interventions or provision of health services and support evidence‐based practice (Churruca et al. 2021; Friedel et al. 2023; Jung et al. 2024). PROMs and PREMs are standardized questionnaires that often consist of multi‐item scales completed directly by the patient. Measures used in genetic counseling (GC) research range from patient empowerment (Costal Tirado et al. 2017; McAllister et al. 2011; Yuen et al. 2020) to measures of counselor‐patient relationship quality, such as working alliance (Erby et al. 2021) or relational empathy (Gonzalez et al. 2024; Zilliacus et al. 2011).

To provide rigorous and meaningful results within and across studies in GC research, PROMs and PREMs require evidence of measurement quality (AERA et al. 2014). In a review of 67 outcome measures (37 non‐genetics‐specific and 30 genetics‐specific measures) used to evaluate clinical genetics services, Payne et al. found that the assessment of measurement quality for PROMs and PREMs in GC research was limited (Payne et al. 2008). In their review, Payne et al. noted that internal consistency reliability, an easily calculated statistic that evaluates how well items measure the same concept, was reported for only 28% of the 67 outcome measures. Although useful, internal consistency reliability reflects only a minimal indicator of measurement quality and does not address measurement validity (i.e., whether a measure actually measures what is intended) or fairness (i.e., lack of bias). Kasparian et al. (2007, page 694) reached a similar conclusion in their review of 25 psychosocial outcome measures used in GC research and called for “increased validation work to be undertaken in genetic counseling research.”

Measurement quality is not an inherent, fixed property of a measurement instrument that generalizes to all purposes, target populations, and contexts (AERA et al. 2014). As illustrated by psychometric meta‐analytic studies such as reliability generalization and validity generalization studies that focus on the heterogeneity of reliability and validity coefficients reported in multiple studies using the same measure (Schmidt and Hunter 1977; Vacha‐Haase 1998), these psychometric coefficients can vary due to many factors. These factors include the characteristics and variability of the sample, length of the questionnaire, where the measure is embedded in the questionnaire (beginning, middle, end), what precedes the scale in the questionnaire, the response scale that is used, etc. Therefore, GC researchers cannot rely exclusively on the psychometric results reported in the original measurement development study or test manual but rather must collect and report psychometric evidence for the measures used in their own GC studies. This requirement of transparency in psychometric reporting is relevant for measures created specifically for GC, as well as for measures adapted from other fields for use in GC research.

### 1.1 Evaluating Validity, Fairness, and Reliability

Validity is more formally defined as the “degree to which evidence and theory support the interpretations of test scores for proposed uses of tests” (AERA et al. 2014). Thus, the validation process is about the meaningfulness of the interpretations and uses of the scores from the measure for a specific purpose, rather than the measure itself. For evidence of validity, the *Standards for Educational and Psychological Testing* (AERA et al. 2014) has identified five major sources of validity evidence. These sources in relation to the current study are: content validity (expert and community partner review), internal structure (confirmatory factor analysis), relations to other variables (e.g., discriminant, concurrent, predictive), response processes (e.g., examined using cognitive interviews), and consequences of testing (e.g., evaluated using community partner input to determine the extent to which the use of PROMs and PREMs improves patient outcomes and minimizes unintended negative consequences such as increasing racial disparities). As noted in the *Standards*, not all sources of validity evidence are relevant for each measure or context in which the measure is used.

For the validity of the scores from multi‐item measures (PROMs and PREMs), Reeve et al. identified content validity and construct validity as two minimum standards (Reeve et al. 2013). Content validity represents the extent to which the multiple items used to measure a construct are relevant, comprehensive, clear to the intended target group, and aligned with the conceptual framework underlying the construct. Content validity can be evaluated using a variety of methods, including expert and participant review, cognitive interviews with members of the target population, and review of items using focus groups. Data from these methods can be analyzed qualitatively (e.g., thematic analysis (Brod et al. 2009)) or quantitatively (e.g., content validity index (Polit and Beck 2006)). When individuals from diverse subgroups are included in the content validation process, issues of fairness can be addressed. As noted in the *Standards* (AERA et al. 2014, page 48), “fairness is a fundamental validity issue and requires attention throughout all stages of test development and use.” Ensuring that individuals from different racial/ethnic groups and/or other minoritized groups interpret items similarly and view the items as relevant for them enhances the fairness of the items.

Construct validity represents the extent to which the selected items measure the theoretical construct that they are intended to measure. Construct validity is a broad term that reflects the unitary theory of validity, which holds that all validity is essentially construct validity (Cronbach and Meehl 1955). Construct validity can be evaluated in many ways, with exploratory factor analysis (EFA) and confirmatory factor analysis (CFA) being two of the most commonly used approaches. EFA is common in the early stages of the instrument development and validation process. CFA is typically used when researchers aim to evaluate if the factor structure of a multi‐item instrument that has been established in previous research is supported within their own sample and context. Evidence from CFA is generally viewed as stronger evidence of validity compared to EFA because CFA operationalizes the theoretical model underlying the measure and provides statistical measures of how well the measurement model fits the empirical data. The measurement model is essentially a set of a priori hypotheses of how many factors (latent variables) underlie the data, how these factors relate to each other, and how the items map onto the latent variables. As a form of construct validity evidence, CFA provides detailed information about the psychometric characteristics (e.g., item factor loadings) of multi‐item scales and model fit (i.e., how well the hypothesized model reproduces the pattern of relationships in the actual data). Model fit measures are described in the Measures section of this paper. Evaluation of model fit is complex and although fit measures are used as general indicators of acceptable model fit, other aspects of the model need to be considered such as potential local model misspecifications and quality of the parameter estimates (e.g., magnitude of the item factor loadings). Evidence of construct validity can also be obtained by examining the pattern of relations between constructs. Cronbach and Meehl, in their seminal paper on construct validation, introduced the nomological network as a way of specifying theoretical propositions of what constructs should be related to each other and what constructs should be unrelated (Cronbach and Meehl 1955).

For the reliability of multi‐item measures (PROMs and PREMs), Reeve et al. reported that internal consistency reliability was necessary to meet the minimum standard for psychometric reporting of reliability (Reeve et al. 2013). Cronbach's alpha is a commonly used method to assess internal consistency reliability. According to Reeve et al.'s (2013) minimum standards, test–retest reliability was not considered a minimum standard due to practical constraints of repeated testing and because patients' true feelings and experiences may fluctuate over time. These fluctuations would violate a key assumption underlying test–retest reliability, which is that individuals' true scores on the construct remain stable over the time interval between measurements.

### 1.2 Purpose and Significance of the Study

The purpose of the present study was to provide validity evidence (content validity, internal structure validity, and relations to other variables), evidence of fairness via expert and community partner input, and reliability (Cronbach's alpha) for the 10 PROMs/PREMs utilized in stage 1 of the Genetic Counseling Processes Result in Outcomes (GC‐PRO) study (listed in Table 1). A key goal was to begin developing a psychometric knowledge base that details how commonly used PROMs and PREMs and their adaptations perform across GC clinical specialties and among patients from diverse racial and ethnic backgrounds across the United States. This study addresses a widely identified gap in GC research, which is the limited reported psychometric information for the measures used in GC research. By providing detailed psychometric information for the 10 measures administered to a large sample of patients in a GC context (*n* = 453), this study offers a foundation for improving the quality of these instruments and serves as a guide for researchers in selecting appropriate measures.

**TABLE 1:** Overview of measures.

Scale	Underlying constructs	Number of items	Response scale	Sample item
**Patient‐reported experience measures (measured post‐visit only)**				
Genetic Counseling Satisfaction^a^	Satisfaction with: length of time spent; counselor's understanding of and empathy toward patient's stress; helpfulness and value of session; feelings of reassurance	6	1–5	The genetic counseling visit was valuable to me
Patient‐Centered Communication	Experience of care that is respectful of and responsive to a patient's preferences and values	7	1–4	How often did the genetic counselor make sure you understood the things you needed to do to take care of your health?
Health Care Climate Questionnaire	Perception of provider's autonomy support versus controlling nature	6	1–7	The genetic counselor tries to understand how I see things before suggesting a new way to do things
State‐Based Information Overload^a^	Information overload (e.g., feeling overwhelmed, stressed, or confused) due to communication of uncertainty and/or high amounts of information	5	1–5	What we talked about in the visit left me feeling overloaded
Working Alliance Inventory^a^	Quality of working alliance (e.g., tasks, bonds, and goals) involving the counselor and client participating collaboratively in the counseling process	10	1–7	The genetic counselor and I are working toward a plan we both agree on
CollaboRATE Shared Decision Making	Shared decision‐making (e.g., explanation of health issue, elicitation of patient preferences, integration of preferences)	3	1–11	How much effort did the genetic counselor make to include what matters most to you in choosing what to do next?
CAHPS Health Literacy Supplemental^a^	Perceived health literacy demand of appointment	3	1–7	The genetic counselor used a lot of medical words I did NOT understand
**Patient‐reported outcome measures (measured pre‐ and post‐visit)**				
Genetic Counseling Outcome Scale^a^	Empowerment across domains of decisional control, behavioral control, emotional regulation, and hope	17	1–5	I can explain what the health condition means to people in my family who may need to know
Patient Activation Measure^a^	Patient activation (e.g., having the skills, knowledge, and motivation to participate in one's own care)	10	1–7	I am confident that I can tell when I need to go get medical care and when I can handle the health condition myself
SURE Decisional Conflict	Clinically significant decisional conflict when facing health or medical choices	4	0–1	Are you clear about which benefits and risks matter most to you?

^a^ Measure modified from original form (see Tables S1–S24 for exact modifications). All measures were unidimensional (one factor) except for the Working Alliance Inventory, which comprised three factors, and the Genetic Counseling Outcome Scale, which comprised four factors. The Genetic Counseling Outcome Scale was also evaluated as a one‐factor model.

## 2 Methods

This study was approved by the University of Minnesota (UMN) single Institutional Review Board (STUDY000011241) on November 30, 2022. All human subjects (e.g., 453 patient participants and 42 genetic counselor participants) provided informed consent prior to their inclusion in the study. Data reported here are from the patient participants only.

### 2.1 Study Overview

The protocol for the GC‐PRO study has been described in detail elsewhere (Fisher et al. 2024). In brief, this was a multi‐site, multi‐discipline study design in which feedback was first obtained on the measures from Community Advisory Board (CAB) members followed by collection of quantitative survey data from patients before and after GC sessions. The overarching goal of the GC‐PRO study was to connect in‐session counseling processes with patient outcomes and experiences to optimize the quality and efficiency of sessions for diverse patient populations.

### 2.2 Procedures

Prior to their GC session, patient participants were recruited via a multi‐pronged approach involving in‐person recruitment, phone call outreach, electronic health portal messaging, and email outreach. Informed consent was obtained consistent with standard research practices prior to study participation. Consented patient participants completed modified versions of the Genetic Counseling Outcome Scale (GCOS) (McAllister et al. 2011) and the Patient Activation Measure (PAM) (a registered trademark of Insignia Health LLC) (Hibbard et al. 2005), an original version of the SURE measure of Decisional Conflict (Légaré et al. 2010), and demographic questions in REDCap. Immediately after their GC session, patient participants completed these same three PROMs, along with seven PREMs (see Table 1). Participants who completed both pre‐visit and post‐visit questionnaires, along with audio recording of their GC visit, were emailed a $30 gift card.

### 2.3 Measures

The study evaluated 10 measures that captured a variety of constructs related to GC, including satisfaction, empowerment, autonomy support, working alliance, shared decision‐making, activation, decisional conflict, and others (Table 1). The scales contained between 2 and 17 items each, with items having varying response scales (e.g., 1 to 5, 1 to 11; see Table 1). All items used higher scores to represent more positive experiences except for the Information Overload Scale (Jensen et al. 2014) where stronger agreement on the five‐point scale represented greater feelings of being overloaded or overwhelmed with information. The Genetic Counseling Satisfaction Scale (GCSS) (DeMarco et al. 2004) was the only measure developed specifically for a GC context that was used in its original form. The remaining nine measures were either developed specifically for GC but modified for the present study, developed outside of GC and adapted for use in the present study, or developed outside of GC and used in its original form (see Table 1).

Four measures were used in their original form and six measures were modified to varying degrees (see Tables S16–S23 for specific information) to align with the specific contexts and target populations for the GC‐PRO study. All measures consisted of one scale except the modified Working Alliance Inventory (WAI) (Erby et al. 2021), which was specified as a three‐factor model consisting of the subscales of Task, Goal, and Bond, and the modified GCOS (McAllister et al. 2011), which was modeled as a one‐factor model and a four‐factor model. The four‐factor model of the GCOS consisted of Decisional Control, Behavioral Control, Emotional Regulation, and Hope dimensions; the original GCOS‐24 consisted of a fifth dimension, Cognitive Control, but not all items for that dimension were included in the present study given the recommendation of Borle et al. (2023) to remove items 1, 14, and 23 to make the scale more applicable in contexts outside of centralized clinical genetic units, and the remaining excluded items demonstrating both statistical and graphical misfit. The decision to use a one‐factor model (unidimensional) for the GCOS was based on McAllister (2025, 625) that “the GCOS‐24 be used as a unidimensional scale, providing one overall scale score” (McAllister 2025).

### 2.4 Analyses

Psychometric analyses were guided by the *Standards for Educational and Psychological Testing* (AERA et al. 2014), the COnsensus‐based Standards for the selection of health Measurement Instruments (COSMIN) framework (Mokkink et al. 2016), and the minimum standards reported by the International Society for Quality of Life Research (Reeve et al. 2013).

#### 2.4.1 Content Validity and Fairness

Prior to initiating data collection for the main study, we gathered feedback from various experts and community partners to evaluate content validity and fairness of the study measures. Survey items were revised by a psychometrician, statistician, and communication expert, who made minor changes to better capture GC outcomes and patient experiences. Subsequently, surveys were piloted by three non‐participating genetic counselors at UMN, one JUNTOS Latino CAB member, and three Faith Works African American CAB members in May and June 2023. Piloting involved virtual semi‐structured think‐aloud interviews that explored overall acceptability of survey items, scales, and ordering; probed specific items that were flagged by the research team as potentially confusing; and assessed the feasibility of the overall survey logistics. The qualitative responses were then reviewed by the research team to identify broad themes that guided modifications of the measures. All study materials were then professionally translated into Spanish by KUMC JUNTOS Center for Advancing Latino Health through a rigorous and iterative process as described in Fisher et al. (2024).

The goals of these analyses were to assess whether the items were culturally appropriate, understandable, and relevant to the target population. Although quantitative analyses such as multi‐group confirmatory factor analysis and differential item functioning (DIF) analysis are commonly used to evaluate measurement fairness by examining whether items function similarly across subgroups (e.g., racial or ethnic groups), the sample sizes for the subgroups in the present study were insufficient to support these analyses. Consequently, evidence related to fairness was derived from qualitative and procedural approaches, including community engagement and expert review. These methods were used to identify potential sources of bias and to enhance the fairness of the measures for the participants being assessed.

#### 2.4.2 Construct Validity

We used CFA in Mplus 9 (Muthén and Muthén 2026) to evaluate the measurement models underlying the 10 measures used in this study. Confirmatory factor analyses for 8 of the 10 measures (see Table 2) were based on the variance‐covariance matrix of the items and were estimated using maximum likelihood estimation with robust standard errors (MLR). For two of the models that consisted of items that used a four‐category (Patient Centered Communication Scale; PCCS) or a two‐category response scale (SURE), polychoric correlations and tetrachoric correlations, respectively, were used in the analyses; weighted least squares mean and variance adjusted (WLSMV) estimation was used in these analyses. Patients were nested within genetic counselors and therefore robust standard errors were obtained in M*plus* using the Type = Complex command to adjust for both non‐normality and non‐independence in the data.

**TABLE 2:** Summary of reliability analyses and confirmatory factor analyses (*n* = 453).

Scale	Cronbach's alpha	*n*	χ^2^	df	CFI	RMSEA 90% CI	SRMR
Genetic Counseling Satisfaction^a^	0.887	436	10.924	9	0.997	0.022 (0.000, 0.061)	0.018
Patient‐Centered Communication^b^	0.935	432	34.527	14	0.998	0.058 (0.034, 0.083)	0.017
Genetic Counseling Outcome Scale one‐factor (pretest)^a^	0.761	453	658.298	119	0.585	0.100 (0.093, 0.108)	0.088
Genetic Counseling Outcome scale four‐factor (pretest)^a^	Dec. = 0.438	453	608.113	113	0.619	0.098 (0.091, 0.106)	0.084
	Beh. = 0.700						
	Emot. = 0.445						
	Hope = 0.556						
Genetic Counseling Outcome Scale one factor (posttest)^a^	0.833	433	544.428	119	0.723	0.091 (0.083, 0.099)	0.075
Genetic Counseling Outcome Scale four‐factor (posttest)^a^	Dec. = 0.480	433	466.382	113	0.770	0.085 (0.077, 0.093)	0.070
	Beh. = 0.772						
	Emot. = 0.548						
	Hope = 0.650						
Health Care Climate (autonomy support)^a^	0.919	433	23.697	9	0.980	0.061 (0.032, 0.092)	0.018
Patient Activation (pretest)^a^	0.863	453	244.112	35	0.823	0.115 (0.101, 0.129)	0.081
Patient Activation (posttest)^a^	0.905	433	178.880	35	0.875	0.097 (0.084, 0.112)	0.055
State‐Based Information Overload^a^	0.809	436	123.823	5	0.626	0.233 (0.199, 0.270)	0.093
Working Alliance Inventory: three‐factor^a^	Task = 0.851	432	112.321	32	0.949	0.076 (0.061, 0.092)	0.038
	Goal = 0.839						
	Bond = 0.793						
SURE Decisional Conflict (pretest)^b^	0.819	186	3.790	2	0.999	0.069 (0.000, 0.176)	0.035
SURE Decisional Conflict (posttest)^b^	0.599	317	2.553	2	0.998	0.030 (0.000, 0.119)	0.072
CollaboRATE (Shared Decision Making)^a^	0.929	433	—	—	—	—	—
CAHPS—Health Literacy Supplemental^a^	0.689	433	—	—	—	—	—

*Note:* Models with three items (i.e., CollaboRATE and CAHPS Health Literacy) are just identified (0 degrees of freedom) and fit the data perfectly, such that fit indices are not meaningful. Modified versions of the Genetic Counseling Outcome Scale (GCOS) and Patient Activation Measure (PAM) and the original version of the SURE Decisional Conflict Scale (SURE) were administered twice, once before the counseling session and once after the session.

Abbreviations: Beh., behavioral control; CFI, comparative fit index; CI, confidence interval; Dec., decisional control; Emot., emotional regulation; RMSEA, root mean square error of approximation; SRMR, standardized root mean square residual.

^a^ Confirmatory factor analysis models estimated using robust maximum likelihood.

^b^ Confirmatory factor analysis models estimated using robust weighted least squares.

Model fit was evaluated using χ^2^, the root mean square error of approximation (RMSEA), the comparative fit index (CFI), and the standardized root mean square residual (SRMR). Hu and Bentler's (1999) cutoff values of < 0.06 on the RMSEA, < 0.08 on the SRMR, and ≥ 0.95 on the CFI were used as general indicators of acceptable model fit (Brown 2015; Hu and Bentler 1999). A non‐significant chi‐square also indicates acceptable fit but because chi‐square can be inflated by large sample sizes, it typically is not used alone as a measure of model fit. In addition to these statistical criteria, we considered conceptual factors when evaluating the models. Good model fit (i.e., the extent to which the pattern of relationships among the items aligns with the theoretical model underlying the measure) provides an important source of construct validity evidence (internal structural validity).

In addition to using model fit as one indicator of psychometric quality, the standardized item factor loadings from the CFAs, representing the strength of the relationship between an item and a factor, provide another source of information about item quality. Hair et al. have suggested that “standardized loading estimates should be 0.5 or higher, and ideally 0.7 or higher” (Hair et al. 2014). Hair et al.'s argument was that standardized loadings > 0.7 indicate that 49% or more of the variance in an item can be explained by the respective factor.

Building on the previous confirmatory factor analyses, which were conducted separately for each measure, we then ran a comprehensive model that simultaneously included all 12 of the constructs included in this study (nine one‐factor measures and the three factors from the WAI) into one latent variable model analysis. The 71 included items were specified to load on their theoretically relevant latent constructs. We used this comprehensive model to evaluate the correlations between all the latent variables measured after the counseling session. This comprehensive model allowed us to test a set of a priori hypotheses representing theoretically expected associations between specific pairs of constructs. For example, we hypothesized that the GCOS and PAM measures would demonstrate a strong positive association when measured after the counseling visit. This model also allowed us to evaluate whether some constructs may be empirically indistinguishable (i.e., lack discriminant validity). Analyses were conducted in Mplus 9 using the weighted least squares mean‐ and variance adjusted (WLSMV) estimator.

#### 2.4.3 Reliability Analyses

Cronbach's alphas were calculated to assess internal consistency reliability for all of the multi‐item measures. Reliability was estimated at both time points for the three measures administered pre‐ and post‐counseling, and for the seven measures administered only after the counseling session. In the case of the modified GCOS, we examined both the single‐factor modified scale and the four subscales.

## 3 Results

### 3.1 Participants

Patient participants included 430 adult patients and 23 parents/legal guardians of underage patients, totaling 453 participants who completed pre‐ and post‐visit surveys and audio recording of their GC sessions. Patients were recruited from two academic health centers (UMN‐M Health Fairview, *n* = 188 and University of South Florida (USF)‐Tampa General Hospital, *n* = 103) and one telehealth GC company (Genome Medical Inc., *n* = 162). Participants primarily identified as women (*n* = 388) and non‐Hispanic White (*n* = 218) with ages ranging from 18 to 88 years (*M* = 40.6, median = 36.0, SD = 13.5). The modal income category was $150,000 or more (*n* = 132); 297 participants had graduated from college or had postgraduate or professional school training (see Table 3). The post‐visit survey completion rate was 95.6%, which was similar across study enrollment locations.

**TABLE 3:** Characteristics of the study sample and genetic counseling sessions.

Variable	*n*	Valid percent
Participant type		
Adult patient	430	94.9
Parent/legal guardian of underage patient	23	5.1
Age		
18–19	2	0.5
20–29	77	17.7
30–39	189	43.3
40–49	74	17.0
50–59	37	8.5
60–69	39	8.9
70–79	14	3.2
80–89	4	0.9
Race/ethnicity		
Asian	61	13.6
Black/African American	54	12.1
Hispanic	76	17.0
Non‐Hispanic White	218	48.8
Multiracial	38	8.5
Gender		
Woman	388	87.0
Man	58	13.0
Education level		
6th–8th grade	1	0.2
9th–10th grade	0	0.0
11th–12th grade	27	6.0
GED or equivalent	13	2.9
Vocational or trade school	17	3.8
Some college	93	20.8
Graduated college	170	37.9
Postgraduate or professional school	127	28.3
Income		
< $25,000	33	8.3
$25,000 to $49,999	40	10.1
$50,000 to $74,999	57	14.3
$75,000 to $99,999	50	12.6
$100,000 to $124,999	50	12.6
$125,000 to $149,999	36	9.0
$150,000 or more	132	33.2
Length of visit		
< 10 min	2	0.4
10 to 19 min	36	7.9
20 to 29 min	84	18.5
30 to 39 min	132	29.1
40 to 49 min	111	24.5
50 to 59 min	59	13.0
60 to 69 min	23	5.1
70 to 79 min	5	1.1
80 to 89 min	1	0.2
Specialty		
Prenatal	171	37.7
Cancer	147	32.5
Cardiology	96	21.2
General genetics	39	8.6
Mode of service delivery		
Phone	177	39.1
Video	174	38.4
In‐Person	102	22.5
Session		
Pre‐test	366	80.8
Post‐test positive result	43	9.5
Post‐test negative result	27	6.0
Post‐test other/uncertain result	17	3.8
Site		
UMN	188	41.5
Genome Medical	162	35.8
USF	103	22.7

*Note:* Missing data for age (*n* = 17); race/ethnicity (*n* = 4 prefer not to answer and *n* = 2 did not respond to the question); gender (*n* = 5, other gender identity [e.g., gender fluid, non‐binary, two‐spirit, etc.]); education level (*n* = 5 prefer not to answer), income (*n* = 11 I don't know and *n* = 42 prefer not to answer).

### 3.2 GC Sessions

GC sessions that were part of the GC‐PRO study ranged from 4 to 86 min (*M* = 37.6, median = 36.0, SD = 13.5) and were delivered via phone (*n* = 177), video (*n* = 174), or in‐person (*n* = 102). Most of the sessions were pre‐test sessions that involved counseling about the option to do genetic testing (*n* = 366), while 87 sessions were post‐test sessions that involved counseling about test results.

### 3.3 Descriptives Analyses

Descriptive statistics for the items in each of the 10 measures are summarized in Tables S1–S15. Positive participant responses were observed for most of the measures with mean scores at the high end of the scales, suggesting potential ceiling effects, particularly among several PREMs.

The three PROMs were administered before and after counseling sessions to evaluate their ability to detect change following the counseling session. Scores on the Patient Activation Measure (a registered trademark of Insignia Health LLC), Genomics Outcomes Scale, and SURE Scale for Decisional Conflict improved significantly from pre‐ to post‐counseling (*p* < 0.001). Cohen's *d* effect sizes indicated moderate to large changes (*d* = 0.59, 0.55, and 0.78, respectively), providing preliminary evidence that the measures were sensitive to change following counseling (see Table S24).

### 3.4 Validity

#### 3.4.1 Content Validity

Using the qualitative input from the expert panels and community partners, the research team identified several themes related to the appropriateness and clarity of the measures. These themes included: (a) recommendations for actionable and feasible modifications, (b) concerns about time to complete surveys, (c) perceptions of question/wording literacy demands, (d) instructions or questions that were confusing or difficult to answer/understand, (e) usability of various scales/formats (5‐point, 7‐point), (f) emotional responses elicited by particular items, and (g) cultural/sensitivity concerns with particular questions. Based on the qualitative feedback and expert review, items that were not appropriate for the current context and target groups were removed (Tables S16–S23). Additional context and/or clarity was added for items that were unclear to participants or raised questions about cultural sensitivity (Tables S16–S23). By systematically addressing the issues raised during the qualitative review process, we strengthened the content validity and fairness of the measures included in the study.

#### 3.4.2 Internal Structure Validity

Table 2 summarizes the results of the CFAs for the 10 measures evaluated in this study. Using the guidelines for the CFI, RMSEA, and SRMR model fit, which represents one source of validity evidence, was generally good for the GCSS, PCCS, HCCQ, WAI, and the SURE measure of Decisional Conflict, which was measured before and after the counseling sessions. Model fit was generally less than acceptable for the modified version of the GCOS (both the one‐factor and four‐factor models, measured before and after the GC sessions), the PAM (assessed before and after the counseling sessions), and the State‐Based Information Overload Scale. Model fit was not evaluated for the Shared Decision‐Making (CollaboRATE) and Health Literacy scales because each scale consisted of three items, which is a just‐identified model that necessarily produces perfect fit (see Table 2).

Tables S1–S15 provide the standardized item‐factor loadings and standard errors for the items in each of the 10 measures. Consistent with the model fit results, strong standardized item factor loadings greater than 0.70 were observed for the GCSS (*M* loading = 0.765), PCCS (*M* loading = 0.941), HCCQ (*M* loading = 0.825), and the SURE Scales (*M* loadings = 0.891 and 0.852, before and after the counseling sessions, respectively). Mean loadings were also above 0.70 for CollaboRATE (*M* = 0.904) and the three WAI factors of Task (*M* = 0.812), Goal (*M* = 0.798), and Bond (*M* = 0.717). The modified version of the GCOS had the lowest average standardized loadings for the one‐factor model when administered prior to (*M* = 0.418) and immediately after the counseling sessions (*M* = 0.498). The four‐factor modified version of the GCOS also had low average loadings with average loadings of 0.411, 0.496, 0.535, and 0.548 for the Decisional Control, Behavioral Control, Emotional Regulation, and Hope factors, respectively, measured prior to the counseling session. These loadings, on average, were slightly better with the data obtained after the counseling session but were still below 0.70 on average (*M* loadings were 0.456, 0.572, 0.617, and 0.614 for the same four factors). The average loading for the three‐item CAHPS Health Literacy Scale was 0.666 but 2 of the 3 items' loadings were above 0.70. Table 4 provides additional details of the item‐factor loadings.

**TABLE 4:** Summary of standardized factor loadings from individual confirmatory factor analyses.

Scale	Number of items	Range of loadings	Average loading	Number of loadings > 0.70	Percentage of loadings > 0.70
Genetic Counseling Satisfaction Scale	6	0.650–0.863	0.765	4	66.7
Patient‐Centered Communication	7	0.902–0.987	0.941	7	100.0
Genetic Counseling Outcome Scale: one‐factor (pretest)	17	0.072–0.630	0.418	0	0.0
Genetic Counseling Outcome Scale: four‐factor (pretest): decisional control	3	0.261–0.503	0.411	0	0.0
Genetic Counseling Outcome Scale: four‐factor (pretest): behavioral control	8	0.344–0.590	0.496	0	0.0
Genetic Counseling Outcome Scale: four‐factor (pretest): emotional regulation	2	0.515–0.555	0.535	0	0.0
Genetic Counseling Outcome Scale: four‐factor (pretest): hope	4	0.230–0.732	0.548	2	50.0
Genetic Counseling Outcome Scale: one‐factor (posttest)	17	0.229–0.719	0.498	3	17.6
Genetic Counseling Outcome Scale: four‐factor (posttest): decisional control	3	0.350–0.541	0.456	0	0.0
Genetic Counseling Outcome Scale: four‐factor (posttest): behavioral control	8	0.449–0.726	0.572	2	25.0
Genetic Counseling Outcome Scale: four‐factor (posttest): emotional regulation	2	0.569–0.664	0.617	0	0.0
Genetic Counseling Outcome Scale: four‐factor (posttest): hope	4	0.255–0.805	0.614	2	50.0
Health Care Climate Questionnaire	6	0.730–0.920	0.825	6	100.0
Patient Activation Measure (pretest)	10	0.334–0.814	0.611	3	30.0
Patient Activation Measure (posttest)	10	0.473–0.807	0.699	6	60.0
State‐Based Information Overload	5	0.421–0.872	0.661	3	60.0
Working Alliance Inventory: task	3	0.746–0.863	0.812	3	100.0
Working Alliance Inventory: goal	3	0.757–0.834	0.798	3	100.0
Working Alliance Inventory: bond	4	0.568–0.796	0.717	3	75.0
SURE Decisional Conflict (pretest)	4	0.774–0.995	0.891	4	100.0
SURE Decisional Conflict (posttest)	4	0.746–0.963	0.852	4	100.0
CollaboRATE (Shared Decision Making)	3	0.844–0.946	0.904	3	100.0
CAHPS—Health Literacy Supplemental	3	0.341–0.924	0.666	2	66.7

*Note:* The Working Alliance Inventory was evaluated as a three‐factor model. The Genetic Counseling Outcome Scale was evaluated as a one‐factor model and a four‐factor model. Standardized factor loadings were obtained from separate confirmatory factor analyses for each individual measure. Pretest = counseling about the option to do genetic testing. Posttest = sessions that involved counseling about test results.

#### 3.4.3 Construct Validity: Relations With Other Variables

The fit of the 12‐factor CFA model was marginally acceptable, χ^2^ (2564, *N* = 453) = 3774.802, *p* < 0.001 (RMSEA = 0.032 [90% confidence interval = 0.030 to 0.034]; CFI = 0.889, SRMR = 0.078). Table 5 presents all 66 Pearson correlations between the 12 constructs. The correlations between the latent constructs, which take into account measurement error, ranged from −0.903 (GCSS and State‐Based Information Overload) to 0.939 (WAI Goal and Bond subscales).

**TABLE 5:** Correlations between constructs from the 12‐factor confirmatory factor analysis (*n* = 453).

	1	2	3	4	5	6	7	8	9	10	11	12
1. Genetic Counseling Satisfaction												
2. Patient‐Centered Communication	0.644											
3. Genetic Counseling Outcomes Scale	0.663	0.558										
4. Health Care Climate	0.792	0.641	0.614									
5. Patient Activation Measure	0.551	0.477	0.867	0.546								
6. State‐Based Information Overload	−0.903	−0.647	−0.760	−0.754	−0.668							
7. Working Alliance Inventory‐task	0.656	0.638	0.712	0.711	0.634	−0.631						
8. Working Alliance Inventory‐goal	0.740	0.691	0.609	0.819	0.538	−0.662	0.854					
9. Working Alliance Inventory‐bond	0.717	0.695	0.588	0.831	0.504	−0.597	0.761	0.939				
10. SURE Decisional Conflict	0.600	0.632	0.379	0.389	0.365	−0.580	0.364	0.383	0.323			
11. CollaboRATE: Shared Decision Making	0.694	0.606	0.455	0.858	0.422	−0.644	0.638	0.688	0.701	0.406		
12. CAHPS—health literacy supplemental	0.845	0.650	0.587	0.915	0.493	−0.893	0.604	0.715	0.722	0.487	0.769	

*Note:* All constructs were measured post‐counseling and were estimated from a 12‐factor confirmatory factor analysis model. One factor was used to represent the Genetic Counseling Outcomes Scale.

As part of the construct validation process, we hypothesized that the GCOS and PAM, as indicators of patient empowerment and activation, would be positively and strongly related. The correlation of 0.867 (*p* < 0.001) between these two constructs supported this hypothesis and adds to the construct validity evidence of these two measures. Additionally, we hypothesized that GCSS, PCCS, HCCQ, WAI, and CollaboRATE, as patient experience measures, would be positively related. Correlational results add to the validity argument for these measures with correlations ranging from 0.606 (PCCS and CollaboRATE) to 0.858 (HCCQ and CollaboRATE). The average correlation between these patient experience measures was 0.709; all correlations were statistically significant (*p* < 0.001).

It should be noted that four correlations were sufficiently large to raise concerns about the distinctiveness of the underlying constructs. Specifically, two subscales of the Working Alliance Inventory, Goal and Bond, were highly correlated (*r* = 0.939). Other notably large correlations were observed between Health Care Climate and the CAHPS Health Literacy Supplemental (*r* = 0.915), between Genetic Counseling Satisfaction and State‐Based Information Overload (*r* = −0.903), and between State‐Based Information Overload and the CAHPS Health Literacy Supplemental (*r* = −0.893). These findings suggest substantial overlap among these measures.

### 3.5 Reliability

Overall, Cronbach's alpha reliabilities ranged from 0.438 for the three‐item Decisional Control subscale of the GCOS to 0.935 for the seven‐item PCCS (*M* reliability across all the scales was 0.740). Of the 23 scales/subscales, 8 had reliabilities < 0.70. These low reliabilities included three of the four modified GCOS subscales at both pre‐counseling and post‐counseling, SURE (post‐counseling), and CAHPS Health Literacy (see Table 2).

## 4 Discussion

Measurement plays a critical role in quantitative GC research, providing the foundation for evaluating GC interventions and describing patients' experiences and outcomes. Sound measurement can advance theory, practice, and policy in GC. The present study was initiated in response to a critical gap in GC research characterized by the limited reporting of psychometric information for commonly used measures in this field. When researchers report detailed information about the psychometric properties of the items used in their research, and when the results are positive and consistent across studies, these findings increase researchers' confidence in the robustness and quality of the measures. When researchers do not report detailed psychometric information, or when psychometric results fluctuate between studies or are consistently poor, this creates uncertainty in the quality of the measures and ultimately in the validity of the conclusions from the research study. The uncertainty in measurement quality in GC could reduce the potential impact research may have on informing theory, policy, and practice.

One of the strengths of this study was that we evaluated the psychometric properties of a large number and variety of PROMs and PREMs employed within a diverse patient sample (51% of patients identifying as Black, Indigenous, and/or Persons of Color) across multiple clinical specialties and types of healthcare institutions. In the present study, we used four measures in their original form and six others that were modified to varying degrees. Our results illustrate how psychometric properties can vary (either increase or decrease in quality) even when the measures are used in their original form. How much the psychometric results change, and in what direction (improve vs. decrease), can only be determined by calculating the values for each application of the measure and reporting these results as part of the cumulative knowledge base for the measure. For example, the six‐item GCSS was used in its original form and had a strong Cronbach's alpha of 0.887, which was similar to the 0.90 reported by (DeMarco et al. 2004). Similarly, the seven‐item PCCS was unchanged and showed excellent psychometric properties. Specifically, Cronbach's alpha was 0.935 in the present study and was similar to the 0.92 value reported in a study of cancer survivors by Moser et al. (2022). Both Moser et al. and GC‐PRO reported the results of a one‐factor CFA, which indicated excellent model fit and strong standardized factor loadings. In contrast, the SURE Decisional Conflict Scale, which was unchanged, had Cronbach's alpha reliabilities of 0.819 (Pre‐session) and 0.599 (Post‐session) in the present study while Légaré et al. (2010) reported Cronbach's alpha reliabilities of 0.54 for “French‐speaking pregnant women considering prenatal screening for Down syndrome” and 0.65 for English‐speaking patients.

Modifications of the other six measures used in the present study varied from minor wording changes to more substantial changes. For example, for the six‐item version of the HCCQ, the term *doctor* was replaced with *genetic counselor*. Model fit and internal consistency reliability for the HCCQ were excellent. In contrast, measures such as the 17‐item GCOS and the 10‐item PAM were changed more dramatically with several items removed and existing items reworded. For these two measures in particular, the psychometric results were mixed in the present study. The modified GCOS had acceptable reliability for the one‐factor model at the pre‐counseling (*α* = 0.761) and post‐counseling sessions (*α* = 0.833) with values slightly lower than internal consistency (*α* = 0.87) reported by McAllister et al. (2011) for the GCOS‐24. However, model fit from the CFA for the modified GCOS was less than acceptable for both the one‐factor and four‐factor models. Additionally, for the pre‐session administration of the modified GCOS, none of the standardized loadings were > 0.70; for the post‐session administration, only 3 of the 17 loadings were > 0.70. Similar mixed results were found for the PAM, which showed acceptable internal consistency at pre‐ and post‐session administrations (*α*s = 0.863 and 0.905, respectively). Model fit was less than acceptable at the pre‐session administration of the measure and marginal at the post‐session administration. Also, of note, the modified GCOS and PAM were highly correlated (*r* = 0.867), suggesting substantial conceptual and empirical overlap despite varied definitions whereby patient activation refers to patients' knowledge, skills, and confidence to manage their health (Hibbard et al. 2005), while patient empowerment reflects the sense of understanding, control, confidence, and emotional adjustment to a genetic condition (McAllister et al. 2011). Given the overlap it may be unnecessary to include both in the same study.

A second strength of this study is that we expanded the psychometric information reported for these measures to include not only internal consistency, but also content validity, internal structure validity using CFA, and construct validity using the nomological network (i.e., the pattern of empirical and theoretical relationships that is used to evaluate a measure's construct validity). In our review of the measures included in the study, we found limited application of CFA as part of the validation process, and we are therefore unable to compare the robustness of our results with those from other studies. CFA is an important source of evidence of validity because it provides statistical measures of how well the measurement model for multi‐item scales with four or more items fits the empirical data as well as detailed information about how well individual items are performing and possible sources of model misfit (e.g., correlated errors).

We did find that in our study some measures performed well as judged by both CFA model fit and Cronbach's alpha (e.g., GCSS, PCCS, HCCQ). In contrast, the four‐item Decisional Conflict (SURE) demonstrated acceptable model fit in CFA, but internal consistency on the post‐counseling measure was low, consistent with the measure's brevity and its role as an index capturing multiple facets of decisional conflict. The State‐Based Information Overload, an existing measure developed outside of GC, illustrates the complexities of evaluating the psychometric quality of a measure. Model fit from the CFA was not acceptable but internal consistency reliability of the five‐item scale was good (Cronbach's *α* = 0.809). Of the five items, three had acceptable standardized loading > 0.70 based on the CFA. The two lower standardized loadings of 0.421 and 0.455 were for items that needed to be reverse coded. Psychometric details about individual items are not frequently reported in published studies so the psychometric performance of reverse‐coded items would remain unknown to researchers considering using this measure.

The reliability results from this study also highlight the importance of calculating and reporting reliability estimates for every measure, as the reliability of a given measure can vary due to a number of factors, including when it is administered. For instance, the modified GCOS demonstrated higher reliability post‐counseling (*α* = 0.833) compared to pre‐counseling (*α* = 0.761). Similar results were found for the PAM with higher reliability post‐counseling (*α* = 0.905) compared to pre‐counseling (*α* = 0.863). In contrast, for the SURE Decisional Conflict Scale, reliability was better at pre‐counseling (*α* = 0.905) compared to post‐counseling (*α* = 0.599). Many factors can affect the reliability of scores including the variability in the responses to the items. Limited variability on item responses can reduce internal consistency reliability. In the case of the SURE Decisional Conflict Scale, 93% or more of the participants responded at post‐counseling *yes* to the four statements that were dichotomously scored, thus reducing variability in the responses. These results, which reveal limited variability in responses, underscore the importance of including item‐level descriptive statistics. Such statistics provide essential context for interpreting a scale's reliability. Additionally, reporting item‐level descriptive statistics to identify potential ceiling or floor tendencies in responses is critical when evaluating model fit and factor loadings in confirmatory factor analyses, since these effects can alter CFA results (e.g., decrease or increase loadings). For example, for the six‐item Genetic Counseling Satisfaction Scale, all six items had mean scores above 4.0 on the five‐point scale. The concentration of responses in the highest response categories may have affected the observed inter‐item associations and factor loadings, although the direction of this effect cannot be determined from descriptive statistics alone. The large percentage of participants selecting *strongly agree*, the highest value on the scale, also reduces the scale's ability to discriminate among respondents with very high satisfaction levels. Ceiling effects on PREMs like the GCCS are not necessarily problematic because the goal is for all patients to have high levels of satisfaction post‐counseling.

Results of the present study underscore the complexity of evaluating the measurement quality of scores from PROMs and PREMs. This study provides an example of how detailed psychometric information for measures can be reported. These details can be provided within the published articles, Supporting Informational sites provided by journals, or in data repositories such as the Open Science framework. Researchers need to report detailed psychometric information for the measures they use in GC research so that a cumulative, empirical knowledge base can be formed that provides guidance to researchers developing or modifying measures for their own research.

### 4.1 Limitations and Directions for Future Research

The results of this quantitative survey study, despite a relatively large sample size for GC research, had small sample sizes across several participant demographic characteristics including race and ethnicity (see Table 3) and prevented the use of quantitative techniques such as multigroup confirmatory factor analysis (MGCFA) to evaluate measurement invariance (i.e., equality of item characteristics such as item factor loadings across groups by race or ethnicity). With larger sample sizes, MGCFA can be used to detect the presence of noninvariance as a potential source of item bias. We were unable to identify any studies within the GC field that have examined measurement invariance for the PROMs and PREMs we examined in our study but did find invariance tests for measures like the PAM outside of GC (Soejima and Kitao 2023). Because we were unable to formally evaluate measurement invariance across demographic groups or clinical settings, it is possible that setting‐ or population‐specific differences may impact measure performance. Future research would benefit from data sharing and collaborative efforts that enable pooling of psychometric datasets across studies. Such approaches, including integrative data analysis (Curran and Hussong 2009), may increase representation of low‐frequency demographic and clinical subgroups and facilitate more rigorous evaluation of measurement invariance.

In addition to evaluating measurement invariance of measures across demographic groups (e.g., gender, age), evaluation of longitudinal measurement invariance is also critical in repeated measures designs (e.g., pretest‐posttest). When measures such as the GCOS are used at two or more points in time (e.g., before a counseling session and after a counseling session), there is no guarantee that the psychometric properties of the measures (e.g., item factor loadings) will remain the same. If the measurement properties of items change, researchers would not know if the observed changes on the constructs are due to real change on the constructs (i.e., changes due to exposure to an intervention), a measurement artifact (e.g., response‐shift bias), or a combination of these two mechanisms. Tests of longitudinal measurement invariance would be recommended in future research for multi‐item measures administered two or more times (see Brown 2015).

The present study evaluated internal consistency reliability (Cronbach's alpha); however, test–retest reliability of the PROMs was not assessed because the participants' scores were expected to change following genetic counseling, rather than remain stable. Future research could evaluate test–retest reliability for the current measures using repeated administrations over a short time interval for participants whose status would be expected to remain stable.

Although pre‐ and post‐counseling data were collected for the three PROMs and provided preliminary evidence of sensitivity to change, the study was not designed to test responsiveness according to prespecified hypotheses or to establish minimal important differences as recommended by the COSMIN framework (Mokkink et al. 2016). Future research should include anchor‐based methods (e.g., patient‐reported global ratings of improvement) to assess responsiveness and establish meaningful change thresholds.

Results of the current study are based on data collected using a self‐completed questionnaire. With the use of a single method of data collection, correlations between constructs may be inflated due to common‐method bias. Other limitations of self‐report questionnaires are response biases such as social desirability, acquiescence, and response fatigue. It is important to note that the present study is part of a larger sequential explanatory mixed‐method study that includes interviews, observations, and other qualitative methods designed to provide descriptions of participants' experiences and outcomes. Results from these qualitative methods will be used to enrich and contextualize the current questionnaire results.

Given that this is a single study, we do not have sufficient evidence to endorse certain measures over others. However, the following points could guide researchers on what they may wish to consider when choosing measures.

1. *Start with what matters most*. Select the PROM or PREM based first on the outcome that is most important to the research question and, ideally, meaningful to patients, not simply on which instrument has the best psychometric properties.
2. *Distinguish PREMs from PROMs and consider responsiveness to change*. PREMs and PROMs answer different questions: PREMs assess patients' experiences of care, whereas PROMs assess patient outcomes that may change as a result of care received. Strong psychometric performance of a PREM therefore does not substitute for a PROM when the goal is to evaluate the impact of GC on patient outcomes. When assessing change over time, researchers should select PROMs that are appropriate for pre‐post administration and have sufficient responsiveness and range to detect meaningful change.
3. *Consider whether the construct can be meaningfully assessed at the intended time point*. Some PROMs may not be applicable to all patients before GC because the relevant issue has not yet arisen or is not yet recognized. For example, in our study, many participants did not know before GC that they would face a decision, making the decisional‐conflict measure (i.e., SURE) irrelevant at baseline for some participants. This does not necessarily indicate that SURE is a poor measure. Rather, when it is used as a pre‐post outcome measure, researchers may need to ensure that patients understand the decision they may be facing before the initial assessment or accept that the measure may not be applicable to all patients at baseline, resulting in incomplete pre‐post data as was the case in our study.
4. *Minimize respondent burden*. Avoid administering multiple measures that provide substantially redundant information. When constructs are conceptually very similar and measures are highly correlated, researchers can consider selecting the measure that best fits the study purpose rather than routinely administering both. When both longer and shorter versions measure the same construct, researchers should weigh gains in psychometric performance against respondent burden; a shorter measure with somewhat lower, but still acceptable, psychometric performance may be preferable when reducing burden is important.
5. *Use accumulating psychometric evidence*. Measure selection should increasingly be informed by evidence across studies, populations, and GC specialties. A weakness seen once may be sample‐specific; repeated problems with factor structure, reliability, responsiveness, are much stronger evidence that a measure may need refinement or may be less appropriate for a particular purpose.
6. *Treat modified instruments cautiously and continue to evaluate validity across populations and settings*. Deleting poorly performing items does not necessarily resolve underlying measurement problems, and modifications to an instrument may alter its psychometric properties. More broadly, validity is not established once for all uses of a measure; evidence should continue to accumulate across populations, clinical settings, and intended uses.

## 5 Conclusions

The present study used a multi‐faceted approach to evaluate the psychometric properties of 10 PROMs and PREMs commonly used in GC research. The GCSS, PCCS, HCCQ, WAI, and CollaboRATE Shared Decision‐Making demonstrated generally strong psychometric performance across multiple sources of evidence. Psychometric results for the modified versions of the GCOS, PAM, State‐Based Information Overload, and SURE measure of Decisional Conflict were more mixed, with some results providing evidence of good measurement quality while other sources of evidence suggested that further refinement or additional psychometric evidence may be needed.

Ultimately, our results argue against a “one‐size‐fits‐all” approach to outcome measurement in GC research. The appropriateness of using one versus multiple measures likely depends on the constructs of interest and the objectives of a given study. There remains a critical need for well‐validated measures that are robust across diverse clinical GC specialties and patient populations, particularly PROMs that can be measured before and after GC. Notably, the PREMs had better psychometric properties than the PROMs in our study, but these measures only ascertain what people think of the GC session, the genetic counselor's behaviors, or their relationship with the genetic counselor and therefore cannot be used to assess pre‐post changes in outcomes, which may be of importance in establishing the impact and importance of GC services.

A key measurement takeaway from the current study is that the evaluation of measurement quality is complex, with results of different psychometric analyses sometimes yielding different conclusions about quality. These differences can raise questions about the robustness of the measures and serve as an impetus for more research to explain these differences. To build a cumulative knowledge base for the measures used in GC research, researchers need to use multiple lenses to evaluate the validity, reliability, and fairness of the GC measures and report these results so that researchers are in a better position to make decisions about what measures may work best for their participants, context, and purposes.

## Author Contributions

**Robert F. Dedrick:** conceptualization, methodology, validation, formal analysis, writing – original draft, writing – review and editing. **Heather A. Zierhut:** conceptualization, methodology, funding acquisition, project administration, writing – review and editing. **Elena R. Fisher:** methodology, project administration, writing – review and editing. **Deborah Cragun:** conceptualization, methodology, funding acquisition, writing – review and editing.

## Funding

This work was supported by the National Human Genome Research Institute, R01HG011916. National Center for Advancing Translational Sciences, UL1TR002494.

## Disclosure

No artificial intelligence tools were used to develop this manuscript.

## Ethics Statement

This study was approved by the University of Minnesota single Institutional Review Board on November 30, 2022 (STUDY00011241). The research team followed standard ethical research standards and all participants gave their informed consent prior to their inclusion in the study.

## Conflicts of Interest

The authors declare no conflicts of interest.

## Supporting information


**Table S1:** Summary information for the genetic counseling satisfaction scale.
**Table S2:** Summary information for the patient‐centered communication scale.
**Table S3:** Summary information for the genetic counseling outcome scale: one‐factor (pretest).
**Table S4:** Summary information for the genetic counseling outcome scale: four‐factor model (pretest).
**Table S5:** Summary information for the genetic counseling outcome scale: one‐factor (posttest).
**Table S6:** Summary information for the genetic counseling outcome scale: four‐factor (posttest).
**Table S7:** Summary information for the health care climate scale.
**Table S8:** Summary information for the patient activation measure (pretest).
**Table S9:** Summary information for the patient activation measure (posttest).
**Table S10:** Summary information for the state‐based information overload.
**Table S11:** Summary information for the three‐factor working alliance inventory.
**Table S12:** Summary information for the sure scale for decisional conflict (pretest).
**Table S13:** Summary information for the sure scale for decisional conflict (posttest).
**Table S14:** Summary information for the collaborate: shared decision making.
**Table S15:** Summary information for the CAHPS—health literacy supplemental.
**Table S16:** Genetic counseling outcomes scale—modifications to items for the GC‐PRO study.
**Table S17:** Patient activation measure—modifications to items for the GC‐PRO study.
**Table S18:** SURE scale—modifications to items for the GC‐PRO study.
**Table S19:** Genetic counseling satisfaction scale—modifications to items for the GC‐PRO study.
**Table S20:** Patient‑centered communication scale*—*modifications to items for the GC‐PRO study.
**Table S21:** State‐based information overload—modifications to items for the GC‐PRO study.
**Table S22:** CollaboRATE shared decision‐making scale—modifications to items for the GC‐PRO study.
**Table S23:** CAHPS health literacy supplemental—modifications to items for the GC‐PRO study.
**Table S24:** Measures administered before and after the counseling sessions.

## Data Availability

The data that support the findings of this study are available on request from the corresponding author. However, some data are not publicly available due to privacy or ethical restrictions.

## References

[jgc470300-bib-0001] American Educational Research Association (AERA), American Psychological Association (APA), and National Council on Measurement in Education (NCME) . 2014. Standards for Educational and Psychological Testing. American Educational Research Association.

[jgc470300-bib-0002] Borle, K. , J. Austin , and S. Barbic . 2023. “Using Rasch Measurement Theory to Explore the Fitness for Purpose of the Genetic Counseling Outcome Scale: A Tale of Two Scales.” Quality of Life Research 32, no. 3: 895–904. 10.1007/s11136-022-03289-7 36413254

[jgc470300-bib-0003] Brod, M. , L. E. Tesler , and T. L. Christensen . 2009. “Qualitative Research and Content Validity: Developing Best Practices Based on Science and Experience.” Quality of Life Research: An International Journal of Quality of Life Aspects of Treatment, Care and Rehabilitation 18, no. 9: 1263–1278. 10.1007/s11136-009-9540-9. 19784865

[jgc470300-bib-0004] Brown, T. A. 2015. Confirmatory Factor Analysis for Applied Research. 2nd ed. Guilford Publications.

[jgc470300-bib-0005] Churruca, K. , C. Pomare , L. A. Ellis , et al. 2021. “Patient‐Reported Outcome Measures (PROMs): A Review of Generic and Condition‐Specific Measures and a Discussion of Trends and Issues.” Health Expectations: An International Journal of Public Participation in Health Care and Health Policy 24, no. 4: 1015–1024. 10.1111/hex.13254. PMC8369118 33949755

[jgc470300-bib-0006] Costal Tirado, A. , A. M. McDermott , C. Thomas , et al. 2017. “Using Patient‐Reported Outcome Measures for Quality Improvement in Clinical Genetics: An Exploratory Study.” Journal of Genetic Counseling 26, no. 5: 1017–1028. 10.1007/s10897-017-0079-6. PMC5582073 28281044

[jgc470300-bib-0007] Cronbach, L. J. , and P. E. Meehl . 1955. “Construct Validity in Psychological Tests.” Psychological Bulletin 52, no. 4: 281–302. 10.1037/h0040957. 13245896

[jgc470300-bib-0008] Curran, P. J. , and A. M. Hussong . 2009. “Integrative Data Analysis: The Simultaneous Analysis of Multiple Data Sets.” Psychological Methods 14, no. 2: 81–100. 10.1037/a0015914. PMC2777640 19485623

[jgc470300-bib-0009] DeMarco, T. A. , B. N. Peshkin , B. D. Mars , and K. P. Tercyak . 2004. “Patient Satisfaction With Cancer Genetic Counseling: A Psychometric Analysis of the Genetic Counseling Satisfaction Scale.” Journal of Genetic Counseling 13, no. 4: 293–304. 10.1023/B:JOGC.0000035523.96133.bc. PMC3551590 19736695

[jgc470300-bib-0010] Erby, L. H. , T. Wisniewski , K. L. Lewis , C. Hernandez , L. G. Biesecker , and B. B. Biesecker . 2021. “Adaptation of the Working Alliance Inventory for the Assessment of the Therapeutic Alliance in Genetic Counseling.” Journal of Genetic Counseling 30, no. 1: 11–21. 10.1002/jgc4.1378. PMC10150706 33554391

[jgc470300-bib-0011] Fisher, E. R. , D. Cragun , R. F. Dedrick , et al. 2024. “Linking Genetic Counseling Communication Skills to Patient Outcomes and Experiences Using a Community‐Engagement and Provider‐Engagement Approach: Research Protocol for the GC‐PRO Mixed Methods Sequential Explanatory Study.” BMJ Open 14, no. 4: e085472. 10.1136/bmjopen-2024-085472. PMC11029319 38631834

[jgc470300-bib-0012] Friedel, A. L. , S. Siegel , C. F. Kirstein , et al. 2023. “Measuring Patient Experience and Patient Satisfaction—How Are we Doing It and Why Does It Matter? A Comparison of European and U.S. American Approaches.” Health 11, no. 6: 60797. 10.3390/healthcare11060797. PMC10048416 36981454

[jgc470300-bib-0013] Gonzalez, T. , K. Tucker , C. E. Wakefield , et al. 2024. “Comparing Cancer Genetic Counselling Using Telegenetics With In‐Person and Telephone Appointments: Results of a Partially Randomised Patient‐Preference Pilot Study.” Journal of Telemedicine and Telecare 30, no. 6: 949–960. 10.1177/1357633X221112556. 35833346

[jgc470300-bib-0014] Hair, J. , M. Sarstedt , L. Hopkins , and V. G. Kuppelwieser . 2014. “Partial Least Squares Structural Equation Modeling (PLS‐SEM): An Emerging Tool in Business Research.” European Business Review 26, no. 2: 106–121. 10.1108/EBR-10-2013-0128.

[jgc470300-bib-0015] Hibbard, J. H. , E. R. Mahoney , J. Stockard , and M. Tusler . 2005. “Development and Testing of a Short Form of the Patient Activation Measure.” Health Services Research 40, no. 6p1: 1918–1930. 10.1111/j.1475-6773.2005.00438.x. PMC1361231 16336556

[jgc470300-bib-0016] Hu, L. , and P. M. Bentler . 1999. “Cutoff Criteria for Fit Indexes in Covariance Structure Analysis: Conventional Criteria Versus New Alternatives.” Structural Equation Modeling: A Multidisciplinary Journal 6, no. 1: 1–55. 10.1080/10705519909540118.

[jgc470300-bib-0017] Jensen, J. D. , N. Carcioppolo , A. J. King , C. L. Scherr , C. L. Jones , and J. Niederdieppe . 2014. “The Cancer Information Overload (CIO) Scale: Establishing Predictive and Discriminant Validity.” Patient Education and Counseling 94, no. 1: 90–96. 10.1016/j.pec.2013.09.016. 24268921

[jgc470300-bib-0018] Jung, A. , D. Challoumas , L. Pagels , S. Armijo‐Olivo , T. Braun , and K. Luedtke . 2024. “Guidelines for the Development and Validation of Patient‐Reported Outcome Measures: A Scoping Review.” BMJ Evidence‐Based Medicine 29, no. 6: 363–373. 10.1136/bmjebm-2023-112681. 38782559

[jgc470300-bib-0019] Kasparian, N. A. , C. E. Wakefield , and B. Meiser . 2007. “Assessment of Psychosocial Outcomes in Genetic Counseling Research: An Overview of Available Measurement Scales.” Journal of Genetic Counseling 16, no. 6: 693–712. 10.1007/s10897-007-9111-6. 17694397

[jgc470300-bib-0020] Légaré, F. , S. Kearing , K. Clay , et al. 2010. “Are You SURE?: Assessing Patient Decisional Conflict With a 4‐Item Screening Test.” Canadian Family Physician Medecin de Famille Canadien 56, no. 8: e308–e314. PMC2920798 20705870

[jgc470300-bib-0021] McAllister, M. 2025. “Genetic Counseling Outcomes, Patient Empowerment, and the Genetic Counseling Outcome Scale.” In The Oxford Handbook of Genetic Counseling. Oxford University Press. 10.1093/oxfordhb/9780190069964.013.0030.

[jgc470300-bib-0022] McAllister, M. , A. Wood , G. Dunn , S. Shiloh , and C. Todd . 2011. “The Genetic Counseling Outcome Scale: A New Patient‐Reported Outcome Measure for Clinical Genetics Services.” Clinical Genetics 79, no. 5: 413–424. 10.1111/j.1399-0004.2011.01636.x. 21255005

[jgc470300-bib-0023] Mokkink, L. B. , C. A. C. Prinsen , L. M. Bouter , H. C. W. de Vet , and C. B. Terwee . 2016. “The COnsensus‐Based Standards for the Selection of Health Measurement INstruments (COSMIN) and How to Select an Outcome Measurement Instrument.” Brazilian Journal of Physical Therapy 20, no. 2: 105–113. 10.1590/bjpt-rbf.2014.0143. PMC4900032 26786084

[jgc470300-bib-0032] Moser, R. P. , N. Trivedi , A. Murray , R. E. Jensen , G. Willis , and K. D. Blake . 2022. “Patient‐Centered Communication (PCC) Scale: Psychometric Analysis and Validation of a Health Survey Measure.” Plos One 17, no. 12: e0279725. 10.1371/journal.pone.0279725. PMC9803193 36584146

[jgc470300-bib-0024] Muthén, L. K. , and B. O. Muthén . 2026. Mplus (Version 9) [Computer Software]. Muthén and Muthén.

[jgc470300-bib-0025] Payne, K. , S. Nicholls , M. McAllister , R. Macleod , D. Donnai , and L. M. Davies . 2008. “Outcome Measurement in Clinical Genetics Services: A Systematic Review of Validated Measures.” Value in Health: The Journal of the International Society for Pharmacoeconomics and Outcomes Research 11, no. 3: 497–508. 10.1111/j.1524-4733.2007.00259.x. 18489673

[jgc470300-bib-0026] Polit, D. F. , and C. T. Beck . 2006. “The Content Validity Index: Are You Sure You Know What's Being Reported? Critique and Recommendations.” Research in Nursing and Health 29, no. 5: 489–497. 10.1002/nur.20147. 16977646

[jgc470300-bib-0027] Reeve, B. B. , K. W. Wyrwich , A. W. Wu , et al. 2013. “ISOQOL Recommends Minimum Standards for Patient‐Reported Outcome Measures Used in Patient‐Centered Outcomes and Comparative Effectiveness Research.” Quality of Life Research: An International Journal of Quality of Life Aspects of Treatment, Care and Rehabilitation 22, no. 8: 1889–1905. 10.1007/s11136-012-0344-y. 23288613

[jgc470300-bib-0028] Schmidt, F. L. , and J. E. Hunter . 1977. “Development of a General Solution to the Problem of Validity Generalization.” Journal of Applied Psychology 62, no. 5: 529–540. 10.1037/0021-9010.62.5.529.

[jgc470300-bib-0033] Soejima, T. , and M. Kitao . 2023. “Adaptation and Measurement Invariance of the 13‐Item Version of Patient Activation Measure Across Japanese Young Adult Cancer Survivors During and After Treatment: A Cross‐Sectional Observational Study.” Plos One 18, no. 9: e0291821. 10.1371/journal.pone.0291821. PMC10508623 37725596

[jgc470300-bib-0029] Vacha‐Haase, T. 1998. “Reliability Generalization: Exploring Variance in Measurement Error Affecting Score Reliability Across Studies.” Educational and Psychological Measurement 58, no. 1: 6–20. 10.1177/0013164498058001002.

[jgc470300-bib-0030] Yuen, J. , S. Y. Lee , E. Courtney , et al. 2020. “Evaluating Empowerment in Genetic Counseling Using Patient‐Reported Outcomes.” Clinical Genetics 97, no. 2: 246–256. 10.1111/cge.13646. 31571202

[jgc470300-bib-0031] Zilliacus, E. M. , B. Meiser , E. A. Lobb , et al. 2011. “Are Videoconferenced Consultations as Effective as Face‐To‐Face Consultations for Hereditary Breast and Ovarian Cancer Genetic Counseling?” Genetics in Medicine 13, no. 11: 933–941. 10.1097/GIM.0b013e3182217a19. 21799430

