# Supplementary material for: Psychometric Analyses of Commonly Used Patient‐Reported Outcome and Experience Measures in Genetic Counseling Research: Findings From the GC‐PRO Study
Source: J Genet Couns. 2026 Oct 23;35(5):e70300. doi: 10.1002/jgc4.70300 (PMC13691921; doi:10.1002/jgc4.70300)
Supplement: Supplementary file 1 [file JGC4-35-0-s001.docx]

## *Supplemental Materials:*

| Table S1  *Summary Information for the Genetic Counseling Satisfaction Scale* | | | | | | | |
| --- | --- | --- | --- | --- | --- | --- | --- |
| Item | *n* | *M* | *SD* | Skewness | Kurtosis | Standardized  Loading | SE |
| 1. The genetic counselor seemed to understand the stresses I was facing. | 436 | 4.394 | 0.695 | -1.166 | 2.159 | 0.789 | 0.038 |
| 2. The genetic counselor helped me to identify what I needed to know to make decisions. | 434 | 4.445 | 0.654 | -1.263 | 3.036 | 0.863 | 0.020 |
| 3. I felt better about my health after meeting with the genetic counselor. | 435 | 4.094 | 0.868 | -0.756 | 0.426 | 0.689 | 0.034 |
| 4. The genetic counselor was truly concerned about my well-being. | 435 | 4.370 | 0.749 | -1.385 | 2.764 | 0.650 | 0.045 |
| 5. The genetic counseling visit was valuable to me. | 432 | 4.417 | 0.706 | -1.469 | 3.815 | 0.806 | 0.042 |
| 6. The genetic counseling visit was about the right length of time I needed. | 434 | 4.493 | 0.663 | -1.621 | 4.656 | 0.791 | 0.036 |

*Note*. Response scale ranged from 1 (Strongly disagree) to 5 (Strongly agree). SE = Standard error.

| Table S2  *Summary Information for the Patient-Centered Communication Scale* | | | | | | | |
| --- | --- | --- | --- | --- | --- | --- | --- |
| Item | *n* | *M* | *SD* | Skewness | Kurtosis | Standardized  Loading | SE |
| 1. Give you the chance to ask all the health-related questions you had | 432 | 3.91 | .355 | -4.409 | 21.862 | .925 | .022 |
| 2. Give the attention you needed to your feelings and emotions | 432 | 3.86 | .411 | -3.348 | 12.262 | .948 | .013 |
| 3. Involve you in decisions about your health care as much as you wanted | 426 | 3.89 | .391 | -4.518 | 24.291 | .987 | .008 |
| 4. Make sure you understood the things you needed to do to take care of your health | 430 | 3.85 | .469 | -3.725 | 15.372 | .921 | .018 |
| 5. Explain things in a way you could understand | 430 | 3.88 | .405 | -3.874 | 17.320 | .942 | .013 |
| 6. Spend enough time with you | 431 | 3.90 | .331 | -3.686 | 14.010 | .962 | .013 |
| 7. Help you deal with feelings of uncertainty about your health or health care | 431 | 3.80 | .523 | -2.854 | 8.386 | .902 | .021 |

*Note*. Response scale ranged from 1 (Never) to 4 (Always). SE = Standard error.

| *Table S3*  *Summary Information for the Genetic Counseling Outcome Scale: One-Factor* *(Pretest)* | | | | | | | |
| --- | --- | --- | --- | --- | --- | --- | --- |
| Item | *n* | *M* | *SD* | Skewness | Kurtosis | Standardized  Loading | SE |
| 1. I can explain what the health condition means to people in my family who may need to know. | 452 | 4.19 | .770 | -.954 | 1.283 | 0.495 | .049 |
| 2. I understand the impact of the health condition on my child(ren)/any child I may have. | 452 | 4.04 | .914 | -.874 | .472 | 0.404 | .048 |
| 3. When I think about the health condition in my family, I get upset. (R) | 451 | 3.24 | 1.106 | -.118 | -.691 | 0.296 | .058 |
| 4. I don’t know where to go to get the medical help I/my family need(s) for this health condition. (R) | 452 | 3.75 | 1.082 | -.697 | -.205 | 0.450 | .046 |
| 5. I can see that good things have come from having this health condition in my family. | 450 | 2.53 | 1.091 | .185 | -.549 | 0.230 | .044 |
| 6. I can control how this health condition affects my family. | 448 | 2.99 | 1.046 | -.128 | -.597 | 0.373 | .042 |
| 7. I am positive about the future. | 452 | 4.09 | .765 | -.606 | .437 | 0.630 | .040 |
| 8. I am able to cope with having this health condition in my family. | 451 | 3.84 | .814 | -.387 | .107 | 0.615 | .040 |
| 9. I don’t know what could be gained from each of the options available to me. (R) | 449 | 3.24 | .946 | .119 | -.404 | 0.357 | .054 |
| 10. In relation to the health condition in my family, nothing I decide will change the future for my child(ren)/any children I might have. (R) | 450 | 3.18 | 1.115 | -.278 | -.580 | 0.072 | .052 |
| 11. I know how to get the non-medical help I/my family needs (e.g. educational, financial, social support). | 449 | 3.66 | .992 | -.674 | -.011 | 0.509 | .052 |
| 12. I can explain what the health condition means to people outside my family who may need to know (e.g. teachers, social workers). | 452 | 3.84 | .866 | -.701 | .594 | 0.538 | .066 |
| 13. I am hopeful that my child(ren)/any children I might have can look forward to a rewarding family life. | 447 | 4.31 | .698 | -.705 | .070 | 0.438 | .042 |
| 14. I am able to make plans for the future. | 447 | 4.30 | .689 | -.838 | 1.144 | 0.614 | .039 |
| 15. I feel guilty because I (might have) passed this health condition on to my children. (R) | 448 | 3.28 | 1.170 | -.187 | -.695 | 0.270 | .059 |
| 16. I am powerless to do anything about this health condition in my family. (R) | 449 | 3.42 | 1.056 | -.317 | -.453 | 0.420 | .047 |
| 17. I can make decisions about the health condition that may change my child(ren)’s future/the future of any child(ren) I may have. | 446 | 3.74 | .862 | -.624 | .629 | 0.392 | .047 |

*Note*. Response scale ranged from 1 (Strongly disagree) to 5 (Strongly agree). R = Reverse-scored. SE = Standard error.

| *Table S4*  *Summary Information for the Genetic Counseling Outcome Scale*: *Four-Factor Model (Pretest)* | | | | | | | |
| --- | --- | --- | --- | --- | --- | --- | --- |
| Item | *n* | *M* | *SD* | Skewness | Kurtosis | Standardized  Loading | SE |
| Decisional Control |  |  |  |  |  |  |  |
| I don’t know what could be gained from each of the options available to me. (R) | 449 | 3.24 | .946 | .119 | -.404 | .503 | .156 |
| In relation to the health condition in my family, nothing I decide will change the future for my child(ren)/any children I might have. (R) | 450 | 3.18 | 1.115 | -.278 | -.580 | .261 | .092 |
| I can make decisions about the health condition that may change my child(ren)’s future/the future of any child(ren) I may have. | 446 | 3.74 | .862 | -.624 | .629 | .470 | .127 |
| Behavioral Control |  |  |  |  |  |  |  |
| I can explain what the health condition means to people in my family who may need to know. | 452 | 4.19 | .770 | -.954 | 1.283 | .560 | .080 |
| I understand the impact of the health condition on my child(ren)/any child I may have. | 452 | 4.04 | .914 | -.874 | .472 | .446 | .071 |
| I don’t know where to go to get the medical help I/my family need(s) for this health condition. (R) | 452 | 3.75 | 1.082 | -.697 | -.205 | .496 | .045 |
| I can control how this health condition affects my family. | 448 | 2.99 | 1.046 | -.128 | -.597 | .344 | .053 |
| I am able to cope with having this health condition in my family. | 451 | 3.84 | .814 | -.387 | .107 | .576 | .060 |
| I know how to get the non-medical help I/my family needs (e.g. educational, financial, social support). | 449 | 3.66 | .992 | -.674 | -.011 | .511 | .058 |
| I can explain what the health condition means to people outside my family who may need to know (e.g. teachers, social workers). | 452 | 3.84 | .866 | -.701 | .594 | .590 | .086 |
| I am powerless to do anything about this health condition in my family. (R) | 449 | 3.42 | 1.056 | -.317 | -.453 | .446 | .068 |
| Emotional Regulation |  |  |  |  |  |  |  |
| When I think about the health condition in my family, I get upset. (R) | 451 | 3.24 | 1.106 | -.118 | -.691 | .515 | .117 |
| I feel guilty because I (might have) passed this health condition on to my children. (R) | 448 | 3.28 | 1.170 | -.187 | -.695 | .555 | .082 |
| Hope |  |  |  |  |  |  |  |
| I can see that good things have come from having this health condition in my family. | 450 | 2.53 | 1.091 | .185 | -.549 | .230 | .054 |
| I am positive about the future. | 452 | 4.09 | .765 | -.606 | .437 | .713 | .050 |
| I am hopeful that my child(ren)/any children I might have can look forward to a rewarding family life. | 447 | 4.31 | .698 | -.705 | .070 | .517 | .053 |
| I am able to make plans for the future. | 447 | 4.30 | .689 | -.838 | 1.144 | .732 | .041 |

*Note*. Response scale ranged from 1 (Strongly disagree) to 5 (Strongly agree). R = Revere-scored. SE = Standard error.

| Table S5  *Summary Information for the Genetic Counseling Outcome Scale: One-Factor* *(Posttest)* | | | | | | | |
| --- | --- | --- | --- | --- | --- | --- | --- |
| Item | *n* | *M* | *SD* | Skewness | Kurtosis | Standardized  Loading | SE |
| 1. I can explain what the health condition means to people in my family who may need to know. | 431 | 4.35 | .666 | -.976 | 2.077 | .584 | .054 |
| 2. I understand the impact of the health condition on my child(ren)/any child I may have. | 428 | 4.29 | .735 | -.916 | .902 | .555 | .044 |
| 3. When I think about the health condition in my family, I get upset. (R) | 433 | 3.4226 | 1.04931 | -.138 | -.648 | .384 | .042 |
| 4. I don’t know where to go to get the medical help I/my family need(s) for this health condition. (R) | 432 | 4.0556 | .94962 | -1.238 | 1.608 | .475 | .046 |
| 5. I can see that good things have come from having this health condition in my family. | 432 | 2.69 | 1.065 | .157 | -.275 | .245 | .064 |
| 6. I can control how this health condition affects my family. | 433 | 3.29 | 1.049 | -.247 | -.513 | .478 | .050 |
| 7. I am positive about the future. | 431 | 4.24 | .725 | -.872 | 1.554 | .701 | .031 |
| 8. I am able to cope with having this health condition in my family. | 430 | 4.04 | .764 | -.453 | -.023 | .719 | .037 |
| 9. I don’t know what could be gained from each of the options available to me. (R) | 431 | 3.8770 | .89505 | -.655 | .436 | .383 | .047 |
| 10. In relation to the health condition in my family, nothing I decide will change the future for my child(ren)/any children I might have. (R) | 432 | 3.3310 | 1.10227 | -.276 | -.631 | .229 | .050 |
| 11. I know how to get the non-medical help I/my family needs (e.g. educational, financial, social support). | 431 | 3.90 | .957 | -.835 | .340 | .481 | .056 |
| 12. I can explain what the health condition means to people outside my family who may need to know (e.g. teachers, social workers). | 431 | 4.19 | .691 | -.518 | .121 | .670 | .046 |
| 13. I am hopeful that my child(ren)/any children I might have can look forward to a rewarding family life. | 431 | 4.29 | .755 | -.802 | .214 | .579 | .055 |
| 14. I am able to make plans for the future. | 430 | 4.37 | .693 | -1.022 | 1.429 | .707 | .047 |
| 15. I feel guilty because I (might have) passed this health condition on to my children. (R) | 430 | 3.5186 | 1.10661 | -.343 | -.591 | .336 | .047 |
| 16. I am powerless to do anything about this health condition in my family. (R) | 433 | 3.7460 | .94533 | -.596 | .067 | .463 | .056 |
| 17. I can make decisions about the health condition that may change my child(ren)’s future/the future of any child(ren) I may have. | 431 | 3.89 | .842 | -.640 | .592 | .485 | .045 |

*Note*. Response scale ranged from 1 (Strongly disagree) to 5 (Strongly agree). R = Reverse-scored. SE = Standard error.

| Table S6  *Summary Information for the Genetic Counseling Outcome Scale: Four-Factor* *(Posttest)* | | | | | | | |
| --- | --- | --- | --- | --- | --- | --- | --- |
| Item | *n* | *M* | *SD* | Skewness | Kurtosis | Standardized  Loading | SE |
| Decisional Control |  |  |  |  |  |  |  |
| I don’t know what could be gained from each of the options available to me. (R) | 431 | 3.8770 | .89505 | -.655 | .436 | .476 | .059 |
| In relation to the health condition in my family, nothing I decide will change the future for my child(ren)/any children I might have. (R) | 432 | 3.3310 | 1.10227 | -.276 | -.631 | .350 | .087 |
| I can make decisions about the health condition that may change my child(ren)’s future/the future of any child(ren) I may have. | 431 | 3.89 | .842 | -.640 | .592 | .541 | .056 |
| Behavioral Control |  |  |  |  |  |  |  |
| I can explain what the health condition means to people in my family who may need to know. | 431 | 4.35 | .666 | -.976 | 2.077 | .654 | .052 |
| I understand the impact of the health condition on my child(ren)/any child I may have. | 428 | 4.29 | .735 | -.916 | .902 | .611 | .046 |
| I don’t know where to go to get the medical help I/my family need(s) for this health condition. (R) | 432 | 4.0556 | .94962 | -1.238 | 1.608 | .480 | .046 |
| I can control how this health condition affects my family. | 433 | 3.29 | 1.049 | -.247 | -.513 | .462 | .053 |
| I am able to cope with having this health condition in my family. | 430 | 4.04 | .764 | -.453 | -.023 | .706 | .042 |
| I know how to get the non-medical help I/my family needs (e.g. educational, financial, social support). | 431 | 3.90 | .957 | -.835 | .340 | .485 | .058 |
| I can explain what the health condition means to people outside my family who may need to know (e.g. teachers, social workers). | 431 | 4.19 | .691 | -.518 | .121 | .726 | .047 |
| I am powerless to do anything about this health condition in my family. (R) | 433 | 3.7460 | .94533 | -.596 | .067 | .449 | .061 |
| Emotional Regulation |  |  |  |  |  |  |  |
| When I think about the health condition in my family, I get upset. (R) | 433 | 3.4226 | 1.04931 | -.138 | -.648 | .664 | .073 |
| I feel guilty because I (might have) passed this health condition on to my children. (R) | 430 | 3.5186 | 1.10661 | -.343 | -.591 | .569 | .056 |
| Hope |  |  |  |  |  |  |  |
| I can see that good things have come from having this health condition in my family. | 432 | 2.69 | 1.065 | .157 | -.275 | .255 | .065 |
| I am positive about the future. | 431 | 4.24 | .725 | -.872 | 1.554 | .740 | .031 |
| I am hopeful that my child(ren)/any children I might have can look forward to a rewarding family life. | 431 | 4.29 | .755 | -.802 | .214 | .656 | .058 |
| I am able to make plans for the future. | 430 | 4.37 | .693 | -1.022 | 1.429 | .805 | .049 |

*Note*. Response scale ranged from 1 (Strongly disagree) to 5 (Strongly agree). R = Reverse-scored. SE = Standard error.

| Table S7  *Summary Information for the Health Care Climate Scale* | | | | | | | |
| --- | --- | --- | --- | --- | --- | --- | --- |
| Item | *n* | *M* | *SD* | Skewness | Kurtosis | Standardized  Loading | SE |
| 1. I feel that the genetic counselor has provided me with choices | 433 | 6.39 | .940 | -2.544 | 8.693 | .746 | .067 |
| 2. I feel understood by the genetic counselor. | 432 | 6.58 | .726 | -3.227 | 17.633 | .920 | .017 |
| 3. The genetic counselor conveys confidence in my ability to make changes or decisions. | 432 | 6.42 | .864 | -2.151 | 6.376 | .792 | .048 |
| 4. The genetic counselor encourages me to ask questions. | 432 | 6.62 | .710 | -3.132 | 14.991 | .874 | .026 |
| 5. The genetic counselor listens to how I would like to do things. | 432 | 6.54 | .791 | -2.514 | 9.002 | .887 | .027 |
| 6. The genetic counselor tries to understand how I see things before suggesting a new way to do things. | 430 | 6.32 | 1.019 | -1.896 | 3.948 | .730 | .049 |

*Note*. Response scale ranged from 1 (Strongly disagree) to 7 (Strongly agree). SE = Standard error.

| Table S8  *Summary Information for the Patient Activation Measure (Pretest)* | | | | | | | |
| --- | --- | --- | --- | --- | --- | --- | --- |
| Item | *n* | *M* | *SD* | Skewness | Kurtosis | Standardized  Loading | SE |
| 1. I am the person who is responsible for managing the health condition. | 452 | 4.17 | .745 | -.775 | .783 | .334 | .054 |
| 2. I am confident that I can take actions that will help prevent or minimize some symptoms or problems associated with the health condition. | 450 | 4.06 | .780 | -.868 | 1.445 | .530 | .035 |
| 3. I know the risks of the health condition. | 452 | 3.79 | .908 | -.551 | -.076 | .666 | .030 |
| 4. I am confident that I can tell when I need to go get medical care and when I can handle the health condition myself. | 452 | 3.89 | .854 | -.782 | .541 | .648 | .035 |
| 5. I am confident I can tell my healthcare provider concerns I have even when he or she does not ask. | 453 | 4.23 | .684 | -.752 | .948 | .470 | .049 |
| 6. I am confident that I can follow through on the plan when I get home. | 452 | 4.30 | .623 | -.427 | -.083 | .471 | .041 |
| 7. I understand the nature and causes of the condition. | 451 | 3.54 | 1.035 | -.430 | -.430 | .730 | .025 |
| 8. I know the different options available for the health condition. | 451 | 3.35 | 1.057 | -.220 | -.674 | .814 | .021 |
| 9. I know how to prevent or deal with further problems with the health condition. | 453 | 3.18 | 1.006 | -.047 | -.555 | .795 | .028 |
| 10. I am confident I can figure out solutions when new situations or problems arise with the health condition. | 451 | 3.72 | .875 | -.423 | .024 | .652 | .035 |

*Note*. Reprinted with permission from Phreesia (PAM® license holder). Response scale ranged from 1 (Strongly disagree) to 5 (Strongly agree). SE = Standard error.

| Table S9  *Summary Information for the Patient Activation Measure (Posttest)* | | | | | | | |
| --- | --- | --- | --- | --- | --- | --- | --- |
| Item | *n* | *M* | *SD* | Skewness | Kurtosis | Standardized  Loading | SE |
| 1. I am the person who is responsible for managing the health condition. | 429 | 4.30 | .713 | -.817 | .484 | .473 | .056 |
| 2. I am confident that I can take actions that will help prevent or minimize some symptoms or problems associated with the health condition. | 429 | 4.19 | .784 | -.939 | 1.100 | .672 | .049 |
| 3. I know the risks of the health condition. | 430 | 4.19 | .719 | -.638 | .283 | .732 | .029 |
| 4. I am confident that I can tell when I need to go get medical care and when I can handle the health condition myself. | 429 | 4.19 | .745 | -.865 | 1.072 | .711 | .030 |
| 5. I am confident I can tell my healthcare provider concerns I have even when he or she does not ask. | 431 | 4.37 | .652 | -1.115 | 2.969 | .618 | .034 |
| 6. I am confident that I can follow through on the plan when I get home. | 429 | 4.33 | .647 | -.602 | .107 | .682 | .039 |
| 7. I understand the nature and causes of the condition. | 432 | 4.00 | .882 | -.760 | .395 | .774 | .027 |
| 8. I know the different options available for the health condition. | 429 | 3.92 | .832 | -.531 | .209 | .807 | .024 |
| 9. I know how to prevent or deal with further problems with the health condition. | 430 | 3.83 | .837 | -.367 | -.275 | .794 | .026 |
| 10. I am confident I can figure out solutions when new situations or problems arise with the health condition. | 431 | 4.07 | .736 | -.528 | .358 | .722 | .032 |

*Note*. Reprinted with permission from Phreesia (PAM® license holder). Response scale ranged from 1 (Strongly disagree) to 5 (Strongly agree). SE = Standard error.

| Table S10  *Summary Information for the State-Based Information Overload* | | | | | | | |
| --- | --- | --- | --- | --- | --- | --- | --- |
| Item | *n* | *M* | *SD* | Skewness | Kurtosis | Standardized  Loading | SE |
| 1. What we talked about in the visit left me feeling overloaded. | 436 | 1.93 | .915 | 1.277 | 2.028 | .811 | .053 |
| 2. I don’t know what I am supposed to do with this information. | 436 | 1.65 | .795 | 1.618 | 3.697 | .746 | .048 |
| 3. I feel overwhelmed by the information. | 433 | 1.79 | .866 | 1.215 | 1.596 | .872 | .027 |
| 4. Most of the information we talked about was helpful. (R) | 436 | 1.5069 | .61584 | 1.338 | 3.906 | .421 | .077 |
| 5. If I wanted, I could explain the main points I learned today to family members or others. (R) | 435 | 1.5816 | .64374 | 1.281 | 4.010 | .455 | .078 |

*Note*. Response scale ranged from 1 (Strongly disagree) to 5 (Strongly agree). SE = Standard error.

| Table S11  *Summary Information for the Three-Factor Working Alliance Inventory* | | | | | | | |
| --- | --- | --- | --- | --- | --- | --- | --- |
| Item | *n* | *M* | *SD* | Skewness | Kurtosis | Standardized  Loading | SE |
| Task Factor | | | | | | | |
| Q1. | 430 | 5.91 | 1.165 | -1.122 | .864 | .826 | .044 |
| Q2. | 427 | 5.57 | 1.336 | -.734 | -.035 | .746 | .037 |
| Q10. | 429 | 6.13 | 1.071 | -1.582 | 2.988 | .863 | .023 |
| Goal Factor | | | | | | | |
| Q4. | 431 | 5.75 | 1.343 | -.950 | .260 | .804 | .024 |
| Q6. | 430 | 5.96 | 1.307 | -1.287 | 1.168 | .757 | .034 |
| Q8. | 428 | 6.23 | .977 | -1.623 | 3.177 | .834 | .035 |
| Bond Factor | | | | | | | |
| Q3. | 430 | 6.02 | 1.128 | -1.231 | 1.294 | .778 | .036 |
| Q5. | 431 | 6.47 | .803 | -2.361 | 8.597 | .796 | .029 |
| Q7. | 428 | 5.82 | 1.193 | -.813 | -.193 | .725 | .039 |
| Q9. | 431 | 5.23 | 1.343 | .117 | -1.385 | .568 | .044 |

*Note*. Items are proprietary with the Society for Psychotherapy Research and cannot be reprinted here. Response scale ranged from 1 (Strongly disagree) to 7 (Strongly agree). SE = Standard error.

| Table S12  *Summary Information for the SURE Scale for Decisional Conflict (Pretest)* | | | | | |
| --- | --- | --- | --- | --- | --- |
| Item | *n* | Percent (%)  No | Percent (%)  Yes | Standardized  Loading | SE |
| 1. Do you feel SURE about the best choice for you? | 186 | 34.9 | 65.1 | .836 | .049 |
| 2. Do you know the benefits and risks of each option? | 186 | 50.5 | 49.5 | .995 | .043 |
| 3. Are you clear about which benefits and risks matter most to you? | 186 | 36.0 | 64.0 | .957 | .031 |
| Do you have enough support and advice to make a choice? | 186 | 18.8 | 81.2 | .774 | .045 |

*Note*. Response scale was 0 (No) or 1 (Yes). SE = Standard error.

| Table S13  *Summary Information for the SURE Scale for Decisional Conflict (Posttest)* | | | | | |
| --- | --- | --- | --- | --- | --- |
| Item | *n* | Percent (%)  No | Percent (%)  Yes | Standardized  Loading | SE |
| 1. Do you feel SURE about the best choice for you? | 316 | 6.3 | 93.7 | .783 | .072 |
| 2. Do you know the benefits and risks of each option? | 316 | 6.0 | 94.0 | .746 | .083 |
| 3. Are you clear about which benefits and risks matter most to you? | 317 | 3.8 | 96.2 | .963 | .107 |
| Do you have enough support and advice to make a choice? | 317 | 0.6 | 99.4 | .917 | .100 |

*Note*. Response scale was 0 (No) or 1 (Yes). SE = Standard error.

| Table S14  *Summary Information for the* CollaboRATE: *Shared Decision Making* | | | | | | | |
| --- | --- | --- | --- | --- | --- | --- | --- |
| Item | *n* | *M* | *SD* | Skewness | Kurtosis | Standardized  Loading | SE |
| 1. How much effort did the genetic counselor make to help you understand the health condition? | 432 | 10.32 | 1.180 | -2.616 | 9.729 | .844 | .032 |
| 2. How much effort did the genetic counselor make to listen to the things that matter most to you about the health condition? | 429 | 10.47 | 1.120 | -3.796 | 20.940 | .946 | .016 |
| 3. How much effort did the genetic counselor make to include what matters most to you in choosing what to do next? | 431 | 10.34 | 1.262 | -3.230 | 14.994 | .923 | .031 |

*Note*. Response scale ranged from 1 (No effort) to 11 (Every effort). SE = Standard error.

| Table S15  *Summary Information for the CAHPS - Health Literacy Supplemental* | | | | | | | |
| --- | --- | --- | --- | --- | --- | --- | --- |
| Item | *n* | *M* | *SD* | Skewness | Kurtosis | Standardized  Loading | SE |
| 1. The genetic counselor used a lot of medical words I did NOT understand. (R) | 431 | 6.0626 | 1.35484 | -1.991 | 3.846 | .733 | .084 |
| 2. The genetic counselor often talked too fast for me to keep up. (R) | 432 | 6.3634 | 1.14792 | -2.991 | 10.150 | .924 | .074 |
| 3. The genetic counselor gave me easy to understand information about my questions or concerns. | 433 | 6.45 | .891 | -3.096 | 14.067 | .341 | .073 |

*Note*. Response scale ranged from 1 (Strongly disagree) to 7 (Strongly agree). SE = Standard error.

| Table S16  *Genetic Counseling Outcomes Scale - modifications to items for the GC-PRO study* | |
| --- | --- |
| **GC-PRO Study** | **Original** **(GCOS-24)** |
| *Not used* | 1. I am clear in my own mind why I am attending the clinical genetics service. |
| I can explain what the health condition means to people in my family who may need to know. | 2. I can explain what the condition means to people in my family who may need to know. |
| I understand the impact of the health condition on my child(ren)/any child I may have. | 3. I understand the impact of the condition on my child(ren)/any child I may have. |
| When I think about the health condition in my family, I get upset. | 4. When I think about the condition in my family, I get upset. |
| I don’t know where to go to get the medical help I/my family need(s) for this health condition. | 5. I don’t know where to go to get the medical help I/my family need(s). |
| I can see that good things have come from having this health condition in my family. | 6. I can see that good things have come from having this condition in my family. |
| I can control how this health condition affects my family. | 7. I can control how this condition affects my family. |
| I am positive about the future. | 8. I feel positive about the future. |
| I am able to cope with having this health condition in my family. | 9. I am able to cope with having this condition in my family. |
| I don’t know what could be gained from each of the options available to me. | 10. I don’t know what could be gained from each of the options available to me. |
| *Not used* | 11. Having this condition in my family makes me feel anxious. |
| *Not used* | 12. I don’t know if this condition could affect my other relatives (brothers,sisters, aunts, uncles, cousins). |
| In relation to the health condition in my family, nothing I decide will change the future for my child(ren)/any children I might have. | 13. In relation to the condition in my family, nothing I decide will change the future for my children/any children I might have. |
| *Not used* | 14. I understand the reasons why my doctor referred me to the clinical genetics service. |
| I know how to get the non-medical help I/my family needs (e.g. educational, financial, social support). | 15. I know how to get the non-medical help I/my family need(s) (e.g.educational, ﬁnancial, social support). |
| I can explain what the health condition means to people outside my family who may need to know (e.g. teachers, social workers). | 16. I can explain what the condition means to people in my family who may need to know. |
| *Not used* | 17. I don’t know what I can do to change how this condition affects me/my children. |
| *Not used* | 18. I don’t know who else in my family might be at risk for this condition. |
| I am hopeful that my child(ren)/any children I might have can look forward to a rewarding family life. | 19. I am hopeful that my children can look forward to a rewarding family life. |
| I am able to make plans for the future. | 20. I am able to make plans for the future. |
| I feel guilty because I (might have) passed this health condition on to my children. | 21. I feel guilty because I (might have) passed this condition on to my children. |
| I am powerless to do anything about this health condition in my family. | 22. I am powerless to do anything about this condition in my family. |
| *Not used* | 23. I understand what concerns brought me to the clinical genetics service. |
| I can make decisions about the health condition that may change my child(ren)’s future/the future of any child(ren) I may have. | 24. I can make decisions about the condition that may change my child(ren)’s future/the future of any child(ren) I may have. |

*Reference:* McAllister, M., Wood, A. M., Dunn, G., Shiloh, S., & Todd, C. (2011). The genetic counseling outcome scale: A new patient‐reported outcome measure for clinical genetics services. Clinical genetics, 79(5), 413-424.

| Table S17  *Patient Activation Measure - modifications to items for the GC-PRO study* | |
| --- | --- |
| **GC-PRO Study** | **Original (PAM-10 and PAM-13)** |
| I am the person who is responsible for managing the health condition. | 1. When all is said and done, I am the person who is responsible for taking care of my health. (PAM-10) |
| *Not used* | 2. Taking an active role in my own health care is the most important thing that affects my health. (PAM-10) |
| I know the risks of the health condition. | 3. I know what each of my prescribed medications do. (PAM-10) |
| I am confident that I can tell when I need to go get medical care and when I can handle the health condition myself. | 4. I am confident that I can tell whether I need to go to the doctor or whether I can take care of a health problem myself. (PAM-10) |
| I am confident I can tell my healthcare provider concerns I have even when he or she does not ask. | 5. I am confident that I can tell a doctor concerns I have even when he or she does not ask. (PAM-10) |
| I am confident that I can follow through on the plan when I get home. | 6. I am confident that I can follow through on medical treatments I may need to do at home. (PAM-10) |
| *Not used* | 7. I have been able to maintain (keep up with) lifestyle changes, like eating right or exercising. (PAM-10) |
| I know how to prevent or deal with further problems with the health condition. | 8. I know how to prevent problems with my health. (PAM-10) // I know how to prevent further problems with my health condition. (PAM-13) |
| I am confident I can figure out solutions when new situations or problems arise with the health condition. | 9. I am confident I can figure out solutions when new problems arise with my health. (PAM-10) |
| *Not used* | 10. I am confident that I can maintain lifestyle changes, like eating right and exercising, even during times of stress. |
| I am confident that I can take actions that will help prevent or minimize some symptoms or problems associated with the health condition. | 11. I am confident I can help prevent or reduce problems associated with my health. (PAM-13) |
| I understand the nature and causes of the condition. | 12. I understand my health problems and what causes them. (PAM-13) |
| I know the different options available for the health condition. | 13. I know what treatments are available for my health problems. (PAM-13) |

*Reference:* Hibbard, J. H., Stockard, J., Mahoney, E. R., & Tusler, M. (2004). Development of the Patient Activation Measure (PAM): conceptualizing and measuring activation in patients and consumers. Health services research, 39(4p1), 1005-1026.

| Table S18  *SURE Scale - modifications to items for the GC-PRO study* | |
| --- | --- |
| **GC-PRO Study** | **Original** |
| Do you feel SURE about the best choice for you | 1. Do you feel sure about the best choice for you? |
| Do you know the benefits and risks of each option? | 2. Do you know the benefits and risks of each option? |
| Are you clear about which benefits and risks matter most to you? | 3. Are you clear about which benefits and risks matter most to you? |
| Do you have enough support and advice to make a choice? | 4. Do you have enough support and advice to make a choice? |

*Reference:* Ferron Parayre, A., Labrecque, M., Rousseau, M., Turcotte, S., & Légaré, F. (2014). Validation of SURE, a four-item clinical checklist for detecting decisional conflict in patients. Medical Decision Making, 34(1), 54-62.

| Table S19  *Genetic Counseling Satisfaction Scale - modifications to items for the GC-PRO study* | |
| --- | --- |
| **GC-PRO Study** | **Original** |
| 8. The genetic counselor seemed to understand the stresses I was facing. | My genetic counselor seemed to understand the stresses I was facing. |
| 9. The genetic counselor helped me to identify what I needed to know to make decisions. | My genetic counselor helped me to identify what I needed to know to make decisions about what would happen to me. |
| 10. I felt better about my health after meeting with the genetic counselor. | I felt better about my health after meeting with my genetic counselor. |
| 11. The genetic counselor was truly concerned about my well-being. | My genetic counselor was truly concerned about my well-being. |
| 12. The genetic counseling visit was valuable to me. | The genetic counseling session was valuable to me. |
| 13. The genetic counseling visit was about the right length of time I needed. | The genetic counseling session was about the right length of time I needed. |

*Reference:* DeMarco TA, Peshkin BN, Mars BD, *et al*. Patient satisfaction with cancer genetic counseling: a psychometric analysis of the genetic counseling satisfaction scale. *J Genet Couns* 2004;13:293–304.

| Table S20  Patient‑Centered Communication Scale *- modifications to items for the GC-PRO study* | |
| --- | --- |
| **GC-PRO Study** | **Original** |
| (Question introduction) How often did the genetic counselor do the following during your visit: | (Question introduction) During the past 12 months, how often did your doctors, nurses or other health care professionals do the following: |
| Give you the chance to ask all the health-related questions you had | 1. Give you the chance to ask all the health-related questions you had |
| Give the attention you needed to your feelings and emotions | 2. Give the attention you needed to your feelings and emotions |
| Involve you in decisions about your health care as much as you wanted | 3. Involve you in decisions about your health care as much as you wanted |
| Make sure you understood the things you needed to do to take care of your health | 4. Make sure you understood the things you needed to do to take care of your health |
| Explain things in a way you could understand | 5. Explain things in a way you could understand |
| Spend enough time with you | 6. Spend enough time with you |
| Help you deal with feelings of uncertainty about your health or health care | 7. Help you deal with feelings of uncertainty about your health or health care |

*Reference:* Moser, R. P., Trivedi, N., Murray, A., Jensen, R. E., Willis, G., & Blake, K. D. (2022). Patient-Centered Communication (PCC) scale: Psychometric analysis and validation of a health survey measure. PLoS One, 17(12), e0279725.

| Supplemental Table  Healthcare Climate Questionnaire *- modifications to items for the GC-PRO study* | |
| --- | --- |
| **GC-PRO Study** | **Original (HCCQ-6)** |
| I feel that the genetic counselor has provided me with choices and options. | 1. I feel that my doctor has provided me choices and options. |
| I feel understood by the genetic counselor. | 2. I feel understood by my doctor. |
| The genetic counselor conveys confidence in my ability to make changes or decisions. | 3. My doctor conveys confidence in my ability to make changes. |
| The genetic counselor encourages me to ask questions. | 4. My doctor encourages me to ask questions. |
| The genetic counselor listens to how I would like to do things. | 5. My doctor listens to how I would like to do things. |
| The genetic counselor tries to understand how I see things before suggesting a new way to do things. | 6. My doctor tries to understand how I see things before suggesting a new way to do things. |

*Reference:* Czajkowska, Z., Wang, H., Hall, N. C., Sewitch, M., & Körner, A. (2017). Validation of the English and French versions of the brief health care climate questionnaire. Health Psychology Open, 4(2), 2055102917730675.

| Table S21  State-Based Information Overload *- modifications to items for the GC-PRO study* | |
| --- | --- |
| **GC-PRO Study** | **Original** |
| What we talked about in the visit left me feeling overloaded. | 1. The cancer news article left me feeling overloaded. |
| I don’t know what I am supposed to do with this information. | 2. I don’t know what I am supposed to do with cancer information like this. |
| I feel overwhelmed by the information. | 3. I feel overwhelmed by the cancer information in the news article |
| *Not used* | 4. It sounded like all the other cancer news articles I have read. |
| *Not used* | 5. I thought it was the cancer news recommendation of the day and that it would probably change tomorrow. |
| Most of the information we talked about was helpful. | 6. It was interesting, but I will probably forget it soon. |
| If I wanted, I could explain the main points I learned today to family members or others. | *No comparison* |

*Reference:* Jensen, J. D., Pokharel, M., Scherr, C. L., King, A. J., Brown, N., & Jones, C. (2017). Communicating uncertain science to the public: How amount and source of uncertainty impact fatalism, backlash, and overload. Risk Analysis, 37(1), 40-51.

| Table S22  CollaboRATE Shared Decision-Making Scale *- modifications to items for the GC-PRO study* | |
| --- | --- |
| **GC-PRO Study** | **Original** |
| How much effort did the genetic counselor make to help you understand the health condition? | 1. How much effort was made to help you understand your health issues? |
| How much effort did the genetic counselor make to listen to the things that matter most to you about the health condition? | 2. How much effort was made to listen to the things that matter most to you about your health issues? |
| How much effort did the genetic counselor make to include what matters most to you in choosing what to do next? | 3. How much effort was made to include what matters most to you in choosing what to do next? |

*Reference:* Barr, P. J., Thompson, R., Walsh, T., Grande, S. W., Ozanne, E. M., & Elwyn, G. (2014). The psychometric properties of CollaboRATE: a fast and frugal patient-reported measure of the shared decision-making process. Journal of medical Internet research, 16(1), e3085.

| Table S23  CAHPS Health Literacy Supplemental *- modifications to items for the GC-PRO study* | |
| --- | --- |
| **GC-PRO Study** | **Original** |
| The genetic counselor used a lot of medical words I did NOT understand. | 1. In the last 6 months, how often did this provider use medical words you did not understand? |
| The genetic counselor often talked too fast for me to keep up. | 2. In the last 6 months, how often did this provider talk too fast when talking with you? |
| The genetic counselor gave me easy to understand information about my questions or concerns. | 3. In the last 6 months, how often did this provider give you easy to understand information about these health questions or concerns? |
| *Not used* | *Additional 17 items in original measure not used |

*Reference:* Weidmer, B. A., Brach, C., & Hays, R. D. (2012). Development and evaluation of CAHPS® survey items assessing how well healthcare providers address health literacy. Medical care, 50(9 Suppl 2), S3.

Supplemental Table 24

*Measures Administered Before and After the Counseling Sessions*

|  |  | Pre-Session | | Post-Session | | Difference Score | |  |  |
| --- | --- | --- | --- | --- | --- | --- | --- | --- | --- |
| Measure | *n* | *M* | *SD* | *M* | *SD* | *M* | *SD* | Cohen’s d for the Difference Score | Pre-Post Session  *r* |
| Patient Activation | 432 | 3.82 | 0.59 | 4.14 | 0.56 | 0.32 | 0.54 | 0.59 | .55 |
|  |  |  |  |  |  |  |  |  |  |
| Genomics Outcomes Scale | 433 | 3.63 | 0.44 | 3.85 | 0.47 | 0.23 | 0.41 | 0.55 | .60 |
|  |  |  |  |  |  |  |  |  |  |
| SURE Scale for Decisional Conflict | 153 | 2.59 | 1.49 | 3.81 | 0.55 | 1.22 | 1.58 | 0.78 | .03 |

*Note*. Response scale for Patient Activation and Genomics Outcomes Scale ranged from 1 (Strongly disagree) to 5 (Strongly agree). The response scale for the SURE Scale for Decisional Conflict ranged from 0 to 4.
